# Personalised health education against health damage of COVID-19 epidemic in the elderly Hungarian population (PROACTIVE-19): protocol of an adaptive randomised controlled clinical trial

**DOI:** 10.1186/s13063-020-04733-0

**Published:** 2020-09-29

**Authors:** Bálint Erőss, Zsolt Molnár, Zsolt Szakács, Noémi Zádori, Lajos Szakó, Szilárd Váncsa, Márk Félix Juhász, Klementina Ocskay, Nóra Vörhendi, Katalin Márta, Andrea Szentesi, Andrea Párniczky, Péter J. Hegyi, Szabolcs Kiss, Mária Földi, Fanni Dembrovszky, Anna Kanjo, Piroska Pázmány, András Varró, Árpád Csathó, Zsuzsanna Helyes, Zoltán Péterfi, László Czopf, István Kiss, Antal Zemplényi, Dóra Czapári, Eszter Hegyi, Dalma Dobszai, Emőke Miklós, Attila Márta, Dominika Tóth, Richard Farkas, Nelli Farkas, Béla Birkás, Erika Pintér, Gábor Pethő, Borbála Zsigmond, Andrea Sárközi, Anikó Nagy, Péter Hegyi

**Affiliations:** 1 Institute for Translational Medicine, Medical School, University of Pécs, Szigeti út 12, Pécs, H-7624 Hungary; 2 Translational Medicine Foundation, Szeged, Hungary; 3 Department of Anaesthesiology and Intensive Therapy, Poznan University for Medical Sciences, Poznan, Poland; 4 János Szentágothai Research Centre, University of Pécs, Pécs, Hungary; 5 Centre for Translational Medicine, Department of Medicine, University of Szeged, Szeged, Hungary; 6 Heim Pál National Pediatric Institute, Budapest, Hungary; 7 Doctoral School of Clinical Medicine, University of Szeged, Szeged, Hungary; 8 Department of Pharmacology and Pharmacotherapy, University of Szeged, Szeged, Hungary; 9 Department of Behavioral Sciences, Medical School, University of Pécs, Pécs, Hungary; 10 Department of Pharmacology and Pharmacotherapy, Medical School, University of Pécs, Pécs, Hungary; 11 Division of Infectious Diseases, 1st Department of Medicine, Medical School, University of Pécs, Pécs, Hungary; 12 Division of Cardiology, First Department of Medicine, Medical School, University of Pécs, Pécs, Hungary; 13 Department of Public Health, Medical School, University of Pécs, Pécs, Hungary; 14 Health Technology Assessment Center, University of Pécs, Pécs, Hungary; 15 Division of Pharmacoeconomics, Department of Pharmaceutics, Faculty of Pharmacy, University of Pécs, Pécs, Hungary; 16 Faculty of Law, University of Szeged, Szeged, Hungary; 17 Institute Bioanalysis, Medical School, University of Pécs, Pécs, Hungary

**Keywords:** COVID-19, SARS-CoV-2, nCov-2019, Public health, Randomised controlled trial, Prevention

## Abstract

**Background:**

Early reports indicate that COVID-19 may require intensive care unit (ICU) admission in 5–26% and overall mortality can rise to 11% of the recognised cases, particularly affecting the elderly. There is a lack of evidence-based targeted pharmacological therapy for its prevention and treatment.

We aim to compare the effects of a World Health Organization recommendation-based education and a personalised complex preventive lifestyle intervention package (based on the same WHO recommendation) on the outcomes of the COVID-19.

**Methods:**

PROACTIVE-19 is a pragmatic, randomised controlled clinical trial with adaptive “sample size re-estimation” design. Hungarian population over the age of 60 years without confirmed COVID-19 will be approached to participate in a telephone health assessment and lifestyle counselling voluntarily. Volunteers will be randomised into two groups: (A) general health education and (B) personalised health education. Participants will go through questioning and recommendation in 5 fields: (1) mental health, (2) smoking habits, (3) physical activity, (4) dietary habits, and (5) alcohol consumption. Both groups A and B will receive the same line of questioning to assess habits concerning these topics. Assessment will be done weekly during the first month, every second week in the second month, then monthly. The composite primary endpoint will include the rate of ICU admission, hospital admission (longer than 48 h), and mortality in COVID-19-positive cases. The estimated sample size is 3788 subjects per study arm. The planned duration of the follow-up is a minimum of 1 year.

**Discussion:**

These interventions may boost the body’s cardiovascular and pulmonary reserve capacities, leading to improved resistance against the damage caused by COVID-19. Consequently, lifestyle changes can reduce the incidence of life-threatening conditions and attenuate the detrimental effects of the pandemic seriously affecting the older population.

**Trial registration:**

The study has been approved by the Scientific and Research Ethics Committee of the Hungarian Medical Research Council (IV/2428- 2 /2020/EKU) and has been registered at clinicaltrials.gov (NCT04321928) on 25 March 2020.

## Background

World Health Organization (WHO) announced the coronavirus disease 2019 (COVID-2019) outbreak pandemic in the morning of 12 March 2020 [1]. At the time of writing this study protocol, there are more than 770,000 confirmed cases with 37,000 fatalities across 178 countries, according to the Center For Systems Science and Engineering (CSSE) at Johns Hopkins University, including 447 cases and 15 deaths in Hungary. The tendency predicts that the epidemic is far from its peak [2].

As often seen in the case of other epidemics, most cases can be asymptomatic or develop only mild symptoms and remain undiagnosed. Therefore, it is difficult to estimate the true incidence and the disease outcomes precisely [3, 4]. However, early reports indicate that it may require intensive care unit (ICU) admission in 5–26%, due to acute respiratory distress syndrome (ARDS) in 17–20%, and overall mortality can rise to 11% of the recognised cases, mostly affecting the elderly [5–8]. These numbers are comparable to the outcomes of earlier coronavirus epidemics [9, 10] and more severe than H1N1 pandemics in 2009 [11].

Significant efforts have been invested in research and development to re-target existing and discover new pharmacological treatments and preventive strategies against COVID-19 [12], as indicated by the number of submitted protocols of the currently recruiting randomised trials on ClinicalTrials.gov. Nevertheless, it must be noted that we lack evidence-based targeted pharmacological therapy for prevention and treatment alike [13]. None of the registered studies investigates the effects of lifestyle interventions in the prevention of poor outcomes in the COVID-19 epidemic. Advanced age and pre-existing comorbidities, such as cancer, cardiovascular disease, or diabetes mellitus, predispose to a more severe disease course and ICU admission [6, 14–16].

The high risk of being infected with COVID-19 as well as the social distancing and quarantining as primary recommendations for the suppression of virus transmission may generate a high level of anxiety and mental stress [17, 18]. In infected patients, better mental health might even have a positive impact on disease progression and survival [19, 20]. Therefore, efforts for better coping with the aversive psychological states caused by the COVID-19 outbreak have high importance in mental health resilience. The role of lifestyle factors and fitness in the severity of COVID-19 has remained unexplored except for two recent studies. The history of smoking is independently associated with disease progression (OR = 14.3, 95% CI 1.6–25.0) in a Chinese cohort of 78 patients [21]. Body mass index was > 25 kg/m^2^ in 88% of patients who died as compared to 19% in survivors in another Chinese cohort of 112 patients [22]. The latter seemingly contradicts the results of a very recent registry analysis of almost 100,000 participants where higher body mass index (indirectly, better nutritional status) proved to be neutral or even preventive although against non-COVID-19 upper airway infections [23]. These suggest that personalised lifestyle interventions via education or counselling could be beneficial for COVID-19 outcomes.

We did not find any complex lifestyle intervention aiming to improve outcomes of epidemic respiratory diseases by a comprehensive literature search. It is likely driven by the difficulty of organising clinical trials with lifestyle interventions. Most problems arise from the following circumstances of epidemics; (1) Exceptionally rapid response is required from the healthcare system. (2) Financial and human resources are often limited and re-routed to manage basic healthcare and public health measures. (3) Often only days are available to set up a trial. (4) The outcomes of the epidemics can only be guessed since the number of influencing variates is almost infinite. (5) We are unable to predict the number of affected cases within a period. (6) Efforts are invested in finding effective pharmacological targets rather in smaller samples. (7) Quarantine is often enforced when lifestyle factors are hard to control, and due to the restriction on personal interactions, informed consent, and data collection cannot be obtained in the usual way. (8) The legislation is not prepared to overcome the difficulties of fast track authorisation and organisation of clinical trials during epidemics. Unsurprisingly, no randomised clinical trial has been performed, to investigate the effects of a multicomponent preventive lifestyle intervention on the outcomes of COVID-19 epidemic.

Our main objective is to evaluate the effects of a personalised multicomponent lifestyle intervention aiming to improve the outcomes of COVID-19 infection in the population over 60 years in a randomised clinical trial. The main hypothesis of PROACTIVE-19 is that the personalised multicomponent lifestyle intervention reduces the rate of our composite outcome consisting of the need for intensive therapy, hospitalisation, and mortality in the COVID-19 population.

## Methods

### Design

The study protocol is structured following Spirit 2013 [24]. PROACTIVE-19 is a pragmatic, randomised controlled clinical trial with adaptive “sample size re-estimation” design. This design allows interim analyses and necessary modifications of the sample size of the ongoing trial to ensure adequate power [25].

### Legislative amendment and ethical approval

In Hungary, Act CLIV of 1997 on Health and Decree No. 23/2002 (of 9 May 2002) of the Minister of Health on Biomedical research on human individuals (as amended) stipulates the procedure for non-interventional investigation, according to which (1) the leader of the investigation or the investigator shall inform the subject both verbally and in writing, before obtaining the consent of the subject to participate in the clinical research, and (2) the participants’ informed consent shall be written. This Act and Decree would not have allowed commencing the clinical trial as it would have amounted to a criminal offence. Based on our request sent to the Prime Minister of Hungary to amend the Decree, the Government of Hungary issued Government Decree No. 63/2020 of 24 March 2020, according to which the new decree amends: (1) in addition to Section 159 of Act CLIV of 1997 on Health, subjects with full disposing capacities can be informed about the non-interventional investigation qualified as clinical research on coronavirus via means of telecommunications; (2) subjects may consent to participate in the clinical research through telecommunications; and (3) subjects may withdraw their consent through telecommunications.

Ethical approval: Scientific and Research Ethics Committee of the Hungarian Medical Research Council (IV/2428- 2 /2020/EKU).

### The trial organisation, committees, and boards

The corresponding centre and designer of the PROACTIVE-19 trial is the Centre for Translational Medicine at the Medical School, University of Pécs, Hungary (coordinating institution and sponsor, www.tm-centre.org).

The Steering Committee (SC) will be led by PH (principal investigator, gastroenterologist, a specialist in internal medicine and clinical pharmacology). SC members will be BE (gastroenterologist, a specialist in internal medicine and primary care), ASz (interdisciplinary unit), ZM (intensive care specialist), and ZH (pharmacologist, a specialist in clinical pharmacology). There will be independent members as well, and the SC will include a patient representative.

The SC will supervise the trial primarily and will make decisions regarding all critical questions (e.g., premature termination of the study, dropouts).

Adjudication Committee (AC): The committee will include a specialist in infectious diseases (ZP), a pharmacologist (AV), and a paediatrician (BZ).

The study was designed by the SC and AC and was supported by the Medical School, University of Pécs. The sponsor had no role in the design of the trial and will have no access to the randomisation codes or the data.

The study will have independent members, including physicians and a safety manager (LC), to comply with current ethical regulations.

### Patient and public involvement

We will perform the assessment and minor modifications in the structure and wording of data collection and the interventions based on the operators’ and participants’ feedback after testing the protocol on 100 potentially eligible subjects. Data of these subjects will not be recorded; only anonymous feedback will be given.

Patients were not included in the recruitment and conduct of the study. Immediately after publications, study results will be disseminated to the population above 60 years of age via the electronic media when, depending on which study arm will better, either general or personal lifestyle intervention will be delivered.

Our interventions do not impose a considerable financial burden on patients; therefore, such compensation will not be required. Volunteering patients, who helped us to test the interventions, claimed that the time and efforts needed to participate in the study and follow the recommendations (of the interventions) are entirely reasonable and acceptable.

### Study population

#### Inclusion and exclusion criteria

The inclusion criteria of our selective primary prevention programme are as follows: (1) age over 60 years (that is, high-risk individuals) and (2) informed consent to participate. The exclusion criteria are as follows: (1) confirmed COVID-19 (active or recovered), (2) hospitalisation at screening for eligibility, and (3) someone was already enrolled in the study from the same community/household (to avoid potential crosstalk between the study arms).

#### Recruitment

The population will be informed about the study and the contact details via social media platforms, newspaper, radio, and television advertisements.

#### Flow and timing

**Fig. 1:**
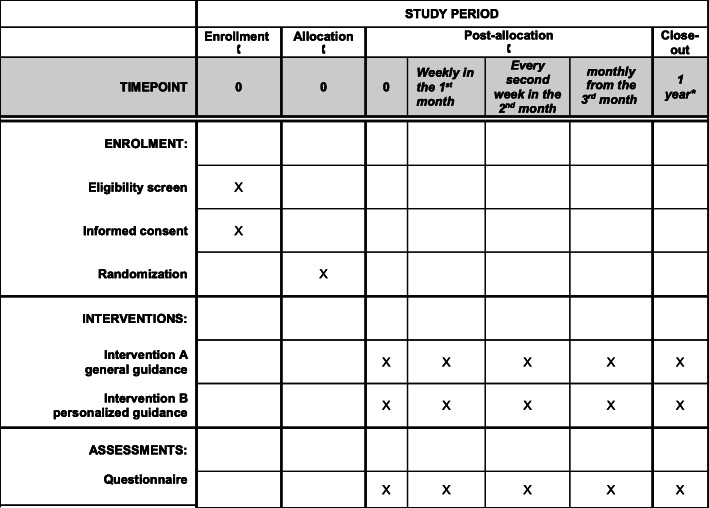
Schedule of enrollment, interventions, and assessments according to the SPIRIT statement. The asterisk indicates that the anticipated finishing date is the end of the pandemic or development of the vaccine, but no more than 1 year from the enrolment of the last participant

A toll-free phone number will be available for all interested in participation. By dialling this number, the participant will be informed about the trial through a pre-recorded voice message, including the study rationale, conditions of participation, the process of the study, and the information on data protection. Willing participants will be redirected to an available operator, who will ascertain eligibility. Following verbal consent and randomisation, the operator will obtain key personal information of the participants and all study-related information (Fig. 1). The allocation will not and cannot be concealed from the operator, but it will be concealed from everyone else (participants, caregivers, outcome assessors).

#### Interventions

Participants will be randomised into two groups: (A) general health education and (B) personalised health education. They will go through questioning and recommendations in 5 domains: (1) mental health, (2) smoking habits, (3) physical activity, (4) dietary habits, and (5) alcohol consumption. Both groups will receive the same line of questioning to assess habits concerning these domains (Suppl. files 1, 2).

Group A: Questioning will be done in the order as mentioned above, followed by a general health education aiming towards improvement of these factors with general recommendations (the expected mean duration is approximately 10 min).

Group B: Questioning will be done in the same structured order, but an assessment of each domain will be followed by personalised recommendations (the expected mean duration is approximately 20 min).

After the first contact, there will be follow-up calls in both groups, with a matching schedule: every week in the first month, every second week in the second month, then monthly. During these encounters, all change in all five domains since the last call will be assessed. The structure, script, and algorithm of the initial and follow-up lifestyle interventions are detailed in Suppl. files 3, 4, respectively.

The operators have received any healthcare education. Before enrolling participants, the operators have to complete a standard training program consisting of seminars on the interventions held by medical professionals, followed by practice of scenarios. The operators will be trained not to give additional healthcare advice, and we will not secure other information sources, including electronic and printed material.

Since standard delivery of the interventions and data collection are essential, the first three and every 50^th^ call of each operator will be assessed. Besides, random calls with various scripts will be made by the study staff to test the operators’ reactions (who are unaware of the test session), followed by detailed assessment and feedback to ensure quality control.

### Outcomes

Based on literature data [5, 26], the primary endpoint will be defined as the composite of any of the following in COVID-19 cases (an accredited laboratory should verify positivity), the rate of:

1. ICU admissions
2. Hospital admissions (longer than 48 h) for the following reasons:
  Arrhythmia (causing hemodynamic instability and requiring continuous monitoring and/or cardiac support, as indicated by mean arterial pressure < 65 mmHg, and/or serum lactate > 2 mmol/L)
  ARDS (severe hypoxaemic respiratory failure indicated by a PaO_2_/FiO_2_ < 300 mmHg according to the Berlin definition) [27]
  Circulatory shock (the requirement of continuous vasopressor support to maintain mean arterial pressure ≥ 65 mmHg and/or serum lactate ≤ 2 mmol/L)
3. Deaths

Secondary endpoints are the following:

1. The number of general practitioner visits
2. The number of emergency, hospital, and intensive care admissions
3. The length of hospitalisation and ICU stay
4. The number of organ dysfunctions and failures (central nervous system, cardiovascular, respiratory, renal, liver, haematological)
5. The measurable lifestyle changes (including physical and mental health)
6. The costs of care

The primary and secondary outcomes will be assessed upon the conclusion of the trial, at least 1 year after the enrolment of the last participant.

### Randomisation and blinding

Computer-generated random sequence randomisation (central) will be performed, after giving informed consent. Due to the expected large sample size, we will use simple randomisation. The allocation ratio will be 1:1. No stratification or blocking will be applied.

In the study, participants will be blinded to the knowledge of the details of differences between the interventions. Everyone else (outcome assessors, caregivers, and data analysts) will be blinded regarding the allocation.

### Sample size calculation, interim, and final analyses

The primary outcome is estimated to occur in 20% of COVID-19-infected cases (≥ 60 years of age) receiving the standard of care based on Chinese reports [5]. Due to the lack of data, we hypothesised that our intervention would result in a 50% risk reduction. Considering one interim analysis on efficacy (with the Pocock correction), 90% power, 5% alpha (superiority design, two-sided), a dropout rate of 20% [28, 29], and assuming 10% incidence of COVID-19 in the target population, the estimated sample size is 3788 (rounded up to 3800) subject per study arm. The calculation was performed by Stata (version 15, Philadelphia, the USA).

We plan to hold three interim analyses: the first for sample size re-estimation at 5% of the target sample size due to the dropout rate, the second for safety assessment at 10% of the target sample size, and the third for efficacy assessment and sample size reestimation at 50% of the target sample size. Early stopping will be executed if (1) safety concerns arise during the interim analysis; (2) the statistical power reaches at least 90% and *p* < 0.05 at the efficacy interim analysis (stopping for benefit); (3) the statistical power does not reach 10%, *p* > 0.05, and the event number does not reach the assumed 10% for the whole population at the efficacy interim analysis (that is, 380 events for the primary outcome; otherwise, the interim analysis is postponed and repeated when the event number reaches 380 events) (stopping for futility); and (4) the consequences of the pandemic make further recruitment or follow-up impossible (stopping for unfeasibility).

In the final analysis, the intention-to-treat analysis will be favoured over per-protocol (or “as-treated”) analysis. We expect a full dataset for the primary endpoint (since the Hungarian Ministry of Interior will provide these data). If for any reason, data will be missing for the primary outcome, we will use available case analysis. The “last observation carried forward” strategy will be followed to impute missing data for other outcomes measured during the study. Missing more than one consecutive interventions after the initial assessment or withdrawal of consent during follow-up results in the dropout of the patients unless hospitalisation is required in the meantime.

In descriptive statistics, the count and percentage will be provided for each treatment arm for binary outcomes. For continuous outcomes, *n*, mean, median, interquartile (Q3–Q1), standard deviation, minimum, and maximum values will be provided for each treatment arm. In a univariate comparative analysis, we will calculate relative risk with 95% confidence interval (CI) when comparing the primary endpoint between two groups (alpha = 5%) with a reference arm using non-repeated intervention complemented with chi-square or Fisher’s exact test (the same strategy will be followed for binary secondary outcomes). For continuous variables, we will use *t* test assuming unequal variances or the Mann-Whitney test. We will perform univariate (Kaplan-Meier and Cox-regression) and multivariate (Cox-regression) survival analysis for binary outcomes. An adjustment will be carried out at least for age, sex, and education. Mixed effect logistic regression will be conducted to estimate the effect of the multicomponent intervention on the outcomes, where the subject IDs will be used as a random subject. The model will be adjusted for changes in smoking habits, alcohol consumption, physical activity, and dietary habits (or body mass index).

All analyses will be carried out with SPSS version 26 and Stata version 15.

### Study duration

The planned starting date of the study is 1 April 2020, and the anticipated finishing date is the end of the pandemic or development of the vaccine, but no more than 1 year from the enrolment of the last participant.

### Data management

#### Data handling

Confidential and anonymous data handling will be performed by the Data Monitoring Committee (DMC). To be able to trace data to an individual subject, a subject identification code list will be used. A Personal Identification Number (PIN) will be generated to identify the data of the participant. This PIN will be present on all forms and documents of each individual. Electronic case report forms (eCRFs) will be used. The Investigator will ensure that the data in the eCRFs are accurate, complete, and legible. Detailed data flow will be described in a Data Management Plan (DMP). Data from completed eCRFs will be validated under the direction of the Data Manager on the DMC according to a Data Cleaning Plan (DCP). Any missing, implausible, or inconsistent recordings in the eCRFs will be referred back to the Investigator using a data query form (DQF). They will be documented for each subject before clean file status is declared. All changes to eCRFs will be recorded.

The DMC will perform an independent assessment of trial-related documents and activities to ensure respect for subjects’ right, safety, and well-being and to guarantee the plausibility of clinical data. The similarity of groups at baseline will also be checked.

Written informed consent had to be replaced, due to the specific circumstances (the need to maintain social distance during the pandemic), by verbal consent obtained during the first call on recruitment. The verbal consent to participate in such clinical research had not been permitted by the law previously. Therefore, the bill was amended on 24 March 2020 upon the request of our study consortium. This amendment enabled us to conduct this trial.

After verbal consent of the subjects, the data will be recorded by the investigator. Clinical research data are processed separately from participants’ data under pseudonyms. Data may only be accessed by persons acting under the authority of the controller and in accordance with the authorisation system established within the controller’s organisational structure, only to the extent and in the manner necessary for the performance of tasks. Personal data are not accessible to third parties.

#### Safety

Due to the nature of the multicomponent moderate-intensity lifestyle intervention, we do not expect serious adverse events. However, minor or moderate adverse events may develop, such as alcohol and nicotine withdrawal, weight change exceeding the optimum, and the need for change in regular medications (antihypertensive or antidiabetic drugs). Participants will be advised to consult their primary care physician if any non-lifestyle-related health issue arises except for COVID-19-related concerns when the call will be transferred to the COVID-19-specific national helpline immediately. If a participant develops a potentially serious health problem, the chairman of the Safety Monitoring Board (LC) will be notified. After the first interim analysis for safety at 10% of the target number, the board will revise the charts of all visits to health facilities and assess if any event is related to the interventions (see, early stopping for safety).

## Discussion

Neither the worldwide climax of the COVID-19 pandemic can be foreseen nor the potential repeated outbreaks [2]. Although efforts of primary prevention (i.e. vaccine development) are promising, it is expected to take 12–18 months from now on [30]. Better lifestyle has its unquestionable advantages not only for infectious but also for common chronic diseases including diabetes mellitus, chronic heart failure or malignant tumours. Considering the recent low numbers of reported cases and the expected trajectory of the epidemic in Hungary, it seems that we are still on time to seek for personalised and easily available public health interventions applicable for the target population.

While in the USA, “remote” consent via telecommunication may be possible, the Hungarian laws have not allowed such initiatives until now. An outbreak imposes new challenges to the process of ethical approval [31]. Most importantly, the instant reaction of both the researchers and the ethical committees is essential, while preserving the validity of scientific content [32].

Based on the results of the current study, such strategies could be introduced in other countries. Lifestyle counselling is expected to reduce mental distress, smoking, and alcohol consumption; increase physical activity; and favourably change the body mass (along with the body composition). As the main results of all these, the interventions may boost the body’s cardiovascular and pulmonary reserve capacities, leading to improved resistance against the damage caused by COVID-19. Consequently, lifestyle changes can reduce the incidence of life-threatening conditions and attenuate the detrimental effects of the pandemic seriously affecting the older population.

### Strengths and limitations

We aim to apply lifestyle interventions considered to be safe in a broad population of subjects exposed at high risk of a severe course of COVID-19. The expected health benefits of the interventions considerably exceed its potential harms. With this study design, we can evaluate the effectiveness of (1) the offer of lifestyle intervention vs (2) that of the actual uptake of or compliance to the lifestyle intervention. We expect that the moderate intensity of the personalised multicomponent lifestyle intervention will maximise the effectiveness and, at the same time, prevents low adherence. In addition to the expected beneficial effects regarding the infection, other protective changes are likely regarding cardiovascular and malignant morbidity and mortality on the long-term. The interventions are easy to be delivered while being affordable and implementable for the vast majority of the population.

We expect that there will be limitations in this study [30]. We define cross-contamination that participants on different arms deliberately and unknowingly communicate with each other, leading to the loss of the true effect of lifestyle interventions. To minimise the risk of cross-contamination, we decided to include only one subject from communities with multiple potential candidate participants. Although we can evaluate the actual uptake of the lifestyle interventions, its validity is uncertain due to the patient-reported nature of the data. We cannot anticipate the climax of the epidemics so that the infection rate of the target population may deviate from the assumed 10%. To overcome this, we use sample size-readjustment adaptive design, which may settle the problem with the unpredictable dropout rate as well (although this method cannot counteract chronological changes in the dropout rate throughout the evolution of the pandemic). All data on secondary outcomes are provided by participants and other, less reliable indirect data sources. We anticipate that volunteers give a representative sample of the target population, but we cannot exclude that our study population will be somewhat better educated and highly motivated. Despite the thorough training of the operators, inter-operator variability may be present.

### Additional information and plans

A follow-up study (PROACTIVE-19 PLUS) is planned to follow up patients, in which blood samples (serum and plasma) from every patient will be stored to analyse immunoglobulins later if required and to build a biobank for a future clinical study. We also intend to publish the study protocol.

## Trial status

Trial registration: The trial has been registered at the clinicaltrials.gov (NCT04321928).

Protocol Version: V1.31.03.2020.

Start of patient recruitment: 01 April 2020.

Anticipated finishing date: the end of the pandemic or development of the vaccine, but no more than 1 year from the enrolment of the last participant.

## Supplementary information

**Additional file 1.**

**Additional file 2.**

**Additional file 3.**

**Additional file 4.**

## Data Availability

Data may only be accessed by persons acting under the authority of the controller and in accordance with the authorisation system established within the controller’s organisational structure, only to the extent and in the manner necessary for the performance of tasks. Personal data are not accessible to third parties. The sponsor will have no access to the database or the randomisation code.
