# Supplementary material for: Personalised health education against health damage of COVID-19 epidemic in the elderly Hungarian population (PROACTIVE-19): protocol of an adaptive randomised controlled clinical trial
Source: Trials. 2020 Sep 29;21:809. doi: 10.1186/s13063-020-04733-0 (PMC7522906; doi:10.1186/s13063-020-04733-0)
Supplement: Supplementary file 1 — Additional file 1. [file 13063_2020_4733_MOESM1_ESM.pdf]

The 5 main domains in detailed

1.) Assessment of mental health:

The high risk of being infected with COVID-19 as well as the social isolation and quarantining as primary recommendations for the suppression of virus transmission may generate a high level of anxiety and mental stress. More specifically, the current evidences strongly indicate that, during the COVID-19 outbreak, disease anxiety, fear, and depression have a highly elevated incidence rate burdening further the health care system and increasing the risk of maladaptive behavioral responses (e.g. failing to comply with the quarantine regulation, and intensifying addictive behavior) [1, 2]. In addition, due to quarantining, everyday social interactions are drastically compromised, which might enhance the feeling of loneliness especially in elderly individuals who are less active in online communities and often no longer live in a family environment. Therefore, efforts for better mental coping with the aversive psychological states caused by the COVID-19 outbreak have high importance in mental health resilience and the compliance with epidemiological instructions. In infected patients, better mental health might even positively impact on survival and disease progression [3, 4].

The questions related to the coronavirus epidemic in will cover in 3 fields: concerns for self, concerns for family, feeling of being overwhelmed on account of news on the epidemic. The answers can be given by a scale ranging from 1-10 points. Higher score indicates greater level of distress.

One question assessess the subjective feeling of being supported, where yes indicates adequate feeling of support and no indicates feeling of being unsupported and/or lonely.

2.) **Smoking**

Current and former smoking habits will be documented in package years. The use of electronic nicotine delivery systems will be recorded. We will encourage all smokers to quit. For participants who seek help, the number of a free-of-charge quitline will be offered [5].

3.) Physical activity

We will record the amount of weekly, regular, moderately intense physical activity. The recommended weekly physical activity based on the WHO guidance will be at least 150 minutes; for additional health benefits we will recommend to increase further to 300 minutes, or more. In order to be beneficial for cardiorespiratory health, all activity should be performed in bouts of at least 10 minutes duration. Additionally, we will encourage everyone to stand up and take a short activity after each hour spent with sitting. In case of participants with physical disability, we will also recommend contacting their physician for a more detailed recommendation.

4.) Dietary habits

Participants of the trial will be asked to provide data on their weight, height and dietary habits and will receive either a detailed-personalized intervention after the assessment (Group B) or a brief, non-

personalized intervention after completing all assessments (Group A). Dietary habits will be assessed using 4 index questions based on a Spanish validated food frequency questionnaire [6].

Based on weight and height data body mass index (BMI) will be calculated. As the BMI-optimum shifts towards the border of normal and overweight ranges with age [7] and studies suggest the protective nature of a BMI of 25-30 kg/m^2 when compared with the normal range, dietary intake reduction will only be recommended if the BMI is greater than 30 kg/m^2 (instead of 25 kg/m^2). 0.5-1 kg reduction per week will be recommended as per the Centers for Disease Control and Prevention (CDC) recommendations [8]. Furthermore, if the individual has a BMI lower than 18.5 kg/m^2 increasing dietary intake will be recommended. In case the participant's BMI is outside the 18.5-30 kg/m^2 range, or the nutritional assessment uncovers unhealthy dietary habits, the participants will receive recommendations according to the WHO's healthy dietary guidelines [9].

5.) Alcohol consumption

Drinking habits of the participants will be assessed, including type, frequency, and the amount of the consumed alcohol. We recommend that participants should avoid alcohol consumption or limit it to 2 units/day and 3 units/day for women and men respectively. We also advise avoiding the consumption of four units at once [10, 11].

REFERENCES

1. Bao, Y., et al., *2019-nCoV epidemic: address mental health care to empower society.* The Lancet, 2020. **395**(10224): p. e37-e38.
2. Xiang, Y.-T., et al., *Timely mental health care for the 2019 novel coronavirus outbreak is urgently needed.* The Lancet Psychiatry, 2020. **7**(3): p. 228-229.
3. Fischbeck, S., et al., *Determinants of illness-specific social support and its relation to distress in long-term melanoma survivors.* BMC public health, 2018. **18**(1): p. 511.
4. Prince, M., et al., *No health without mental health.* The lancet, 2007. **370**(9590): p. 859-877.
5. *Az Emberi Erőforrások Minisztériuma egészségügyi szakmai irányelve a dohányzásról való leszokás támogatásáról*, E.E. Minisztériuma, Editor. 2019: https://tudogyogyasz.hu.
6. Bejar, L.M. and E. Vazquez-Limon, *Is there any alternative to traditional food frequency questionnaire for evaluating habitual dietary intake?* Nutr Hosp, 2017. **34**(4): p. 990-888.
7. Bhaskaran, K., et al., *Association of BMI with overall and cause-specific mortality: a population-based cohort study of 3.6 million adults in the UK.* Lancet Diabetes Endocrinol, 2018. **6**(12): p. 944-953.
8. Nishiura, H., et al., *Estimation of the asymptomatic ratio of novel coronavirus infections (COVID-19).* Int J Infect Dis, 2020.
9. WHO, *Healthy diet.* https://www.who.int/news-room/fact-sheets/detail/healthy-diet, 2018.
10. *Drinking Guidelines: General Population*. 2019.
11. Liu, Z., et al., *Mobile Phone-Based Lifestyle Intervention for Reducing Overall Cardiovascular Disease Risk in Guangzhou, China: A Pilot Study.* International journal of environmental research and public health, 2015. **12**(12): p. 15993-16004.
