# Supplementary material for: Personalised health education against health damage of COVID-19 epidemic in the elderly Hungarian population (PROACTIVE-19): protocol of an adaptive randomised controlled clinical trial
Source: Trials. 2020 Sep 29;21:809. doi: 10.1186/s13063-020-04733-0 (PMC7522906; doi:10.1186/s13063-020-04733-0)
Supplement: Supplementary file 2 — Additional file 2. [file 13063_2020_4733_MOESM2_ESM.pdf]

First call
Stress

English protocol

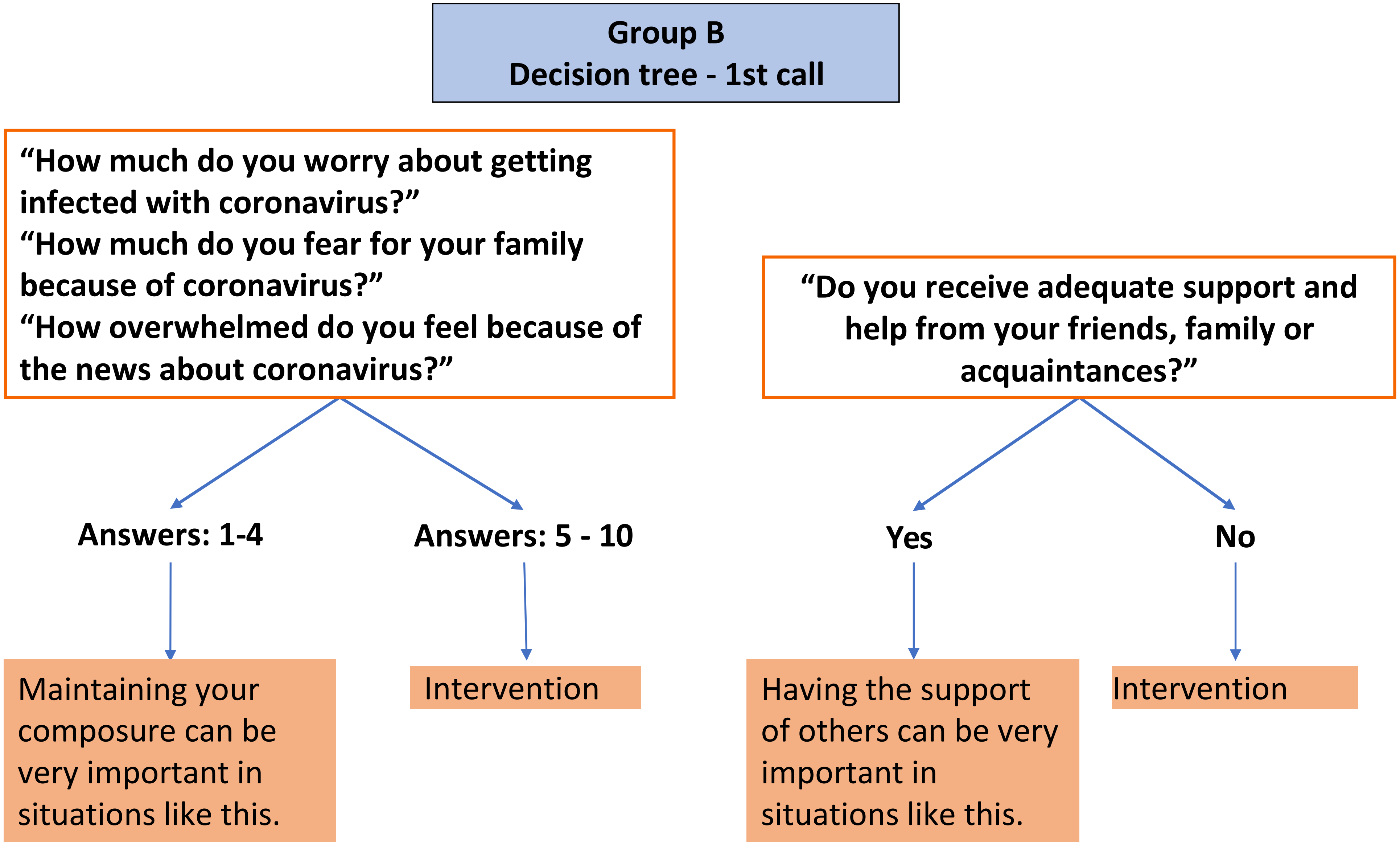
Group B
Decision tree - 1st call
“How much do you worry about getting infected with coronavirus?”
“How much do you fear for your family because of coronavirus?”
“How overwhelmed do you feel because of the news about coronavirus?”
Answers: 1-4
Answers: 5 - 10
Maintaining your composure can be very important in situations like this.
Intervention
“Do you receive adequate support and help from your friends, family or acquaintances?”
Yes
No
Having the support of others can be very important in situations like this.
Intervention

Intervention

“As much as your opportunities allow you to keep up your social connections, stay in touch with your family, friends, and acquaintances. If because of coronavirus related regulations you are required to avoid physical contact with others, try maintaining your social connections through other platforms, e.g. telephone, e-mail, social media (Facebook, TikTok, Skype, Twitter, etc.).

Establish a daily rhythm! Pay attention to your own needs and feelings. Engage in activities that you like and that make you feel calm, for example: watching movies or reading.

Share your thoughts with others! At the same time, you also should pay attention to the ones around you, listen to them, encourage them to share their experiences and feelings.

Constantly following news related to the epidemic can make you feel overwhelmed. Try to follow this news only periodically! Try to avoid all information that is not coming from a reliable source. You should primarily rely on news coming from healthcare professionals, for example the governmental information page: https://koronavirus.gov.hu/. Or dial any of the following informational lines: (06 80 277 455 / 06 80 277 456).
Because of the coronavirus related regulations, our social interactions can become limited easily, anybody can feel alone in a situation like this.
If you are feeling really alone, you should seek professional help. For example, call the *’Magyar Lelki elsősegély* telefonszolgálat’: 116-123”

First call
Smoking

English protocol

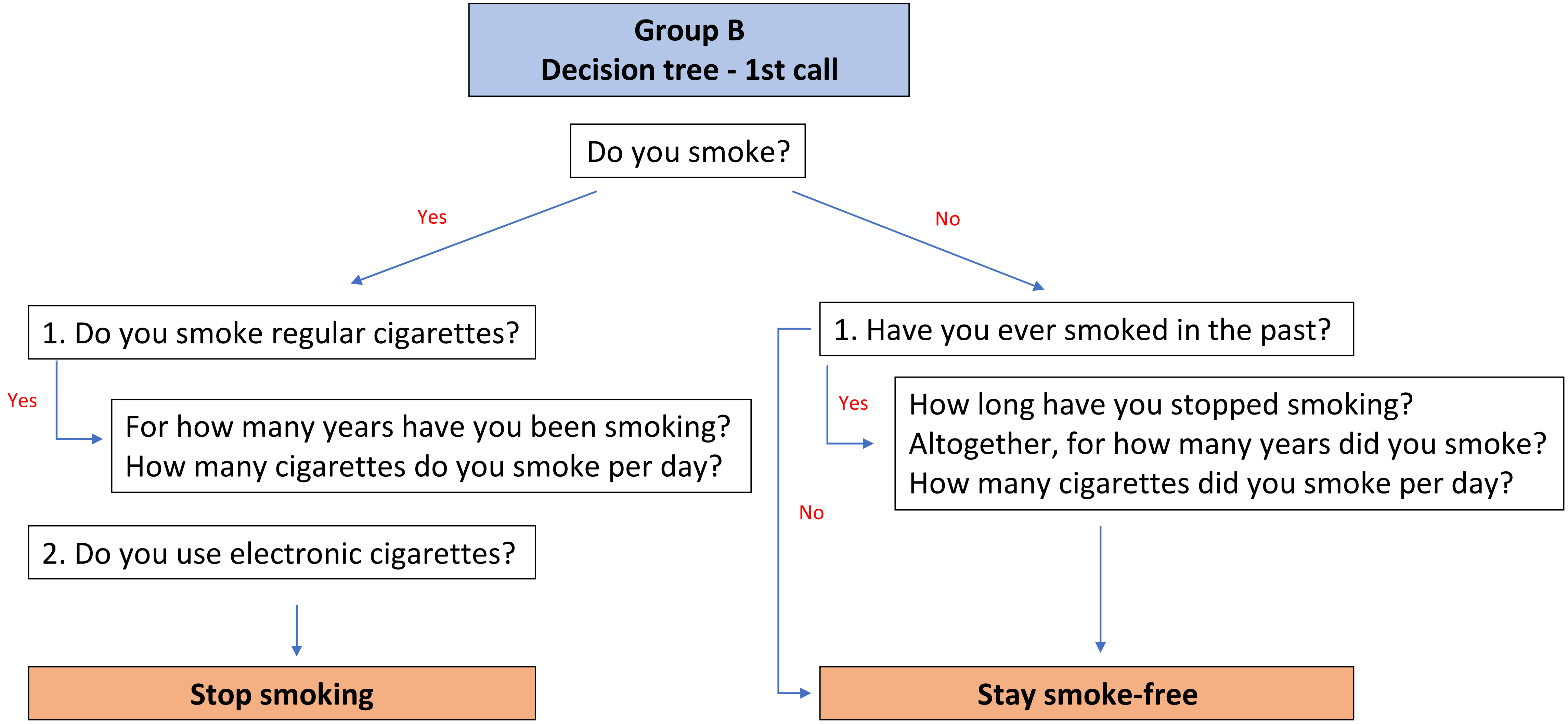
Group B
Decision tree - 1st call
Do you smoke?
Yes
No
1. Do you smoke regular cigarettes?
Yes
For how many years have you been smoking?
How many cigarettes do you smoke per day?
2. Do you use electronic cigarettes?
Stop smoking
1. Have you ever smoked in the past?
Yes
How long have you stopped smoking?
Altogether, for how many years did you smoke?
How many cigarettes did you smoke per day?
No
Stay smoke-free

Stop smoking

“To our knowledge, among the lifestyle factors, tobacco smoking has the greatest impact on the outcome of coronavirus cases. The course of upper airway infections is milder in non-smokers. Accordingly, we can highly recommend that you stop smoking as soon as possible. If you are unable to do this, you might cut down smoking at least. If you need any further support to quit smoking, please let us know now.“

If yes: “To get help with quitting, call 06-80-44-20-44, which is a free service.”

Stay smoke-free

“Very good, keep it up, and stay smoke-free! The course of upper airway infections is milder in non-smokers.”

First call
Physical activity

English protocol

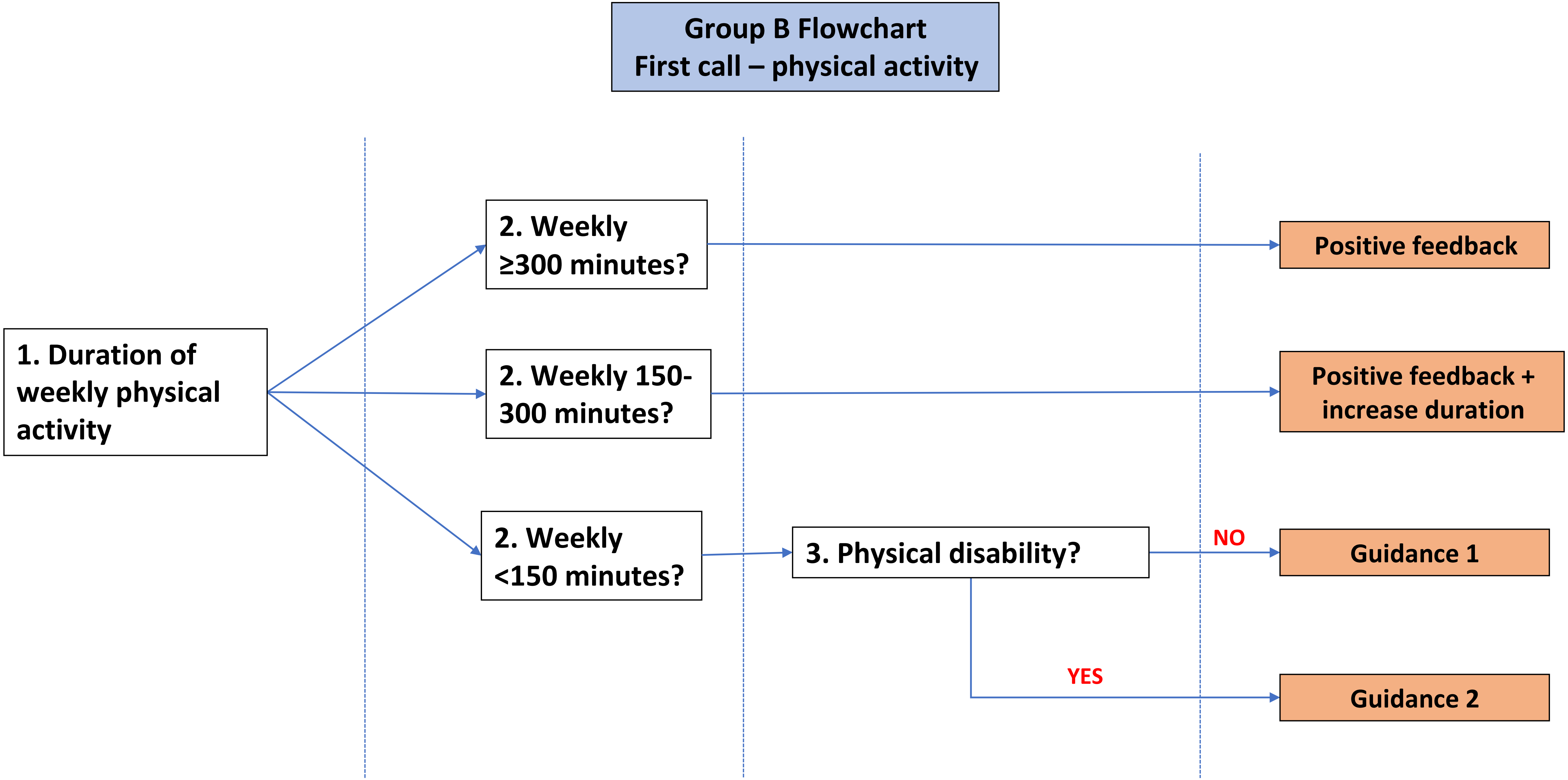
Group B Flowchart
First call – physical activity
1. Duration of weekly physical activity
2. Weekly ≥300 minutes?
2. Weekly 150-300 minutes?
2. Weekly <150 minutes?
3. Physical disability?
NO
YES
Positive feedback
Positive feedback + increase duration
Guidance 1
Guidance 2

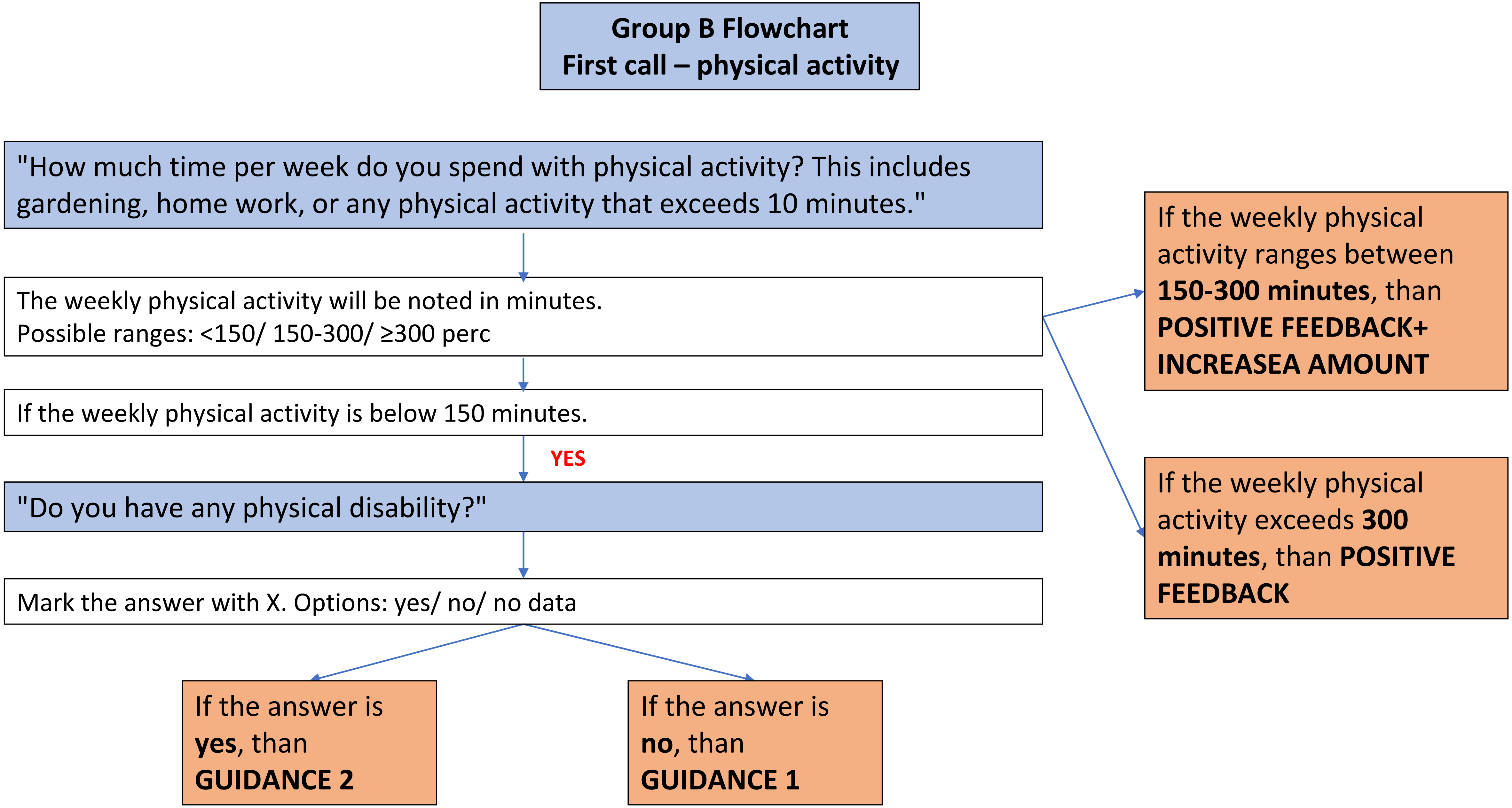
Group B Flowchart
First call – physical activity
"How much time per week do you spend with physical activity? This includes gardening, home work, or any physical activity that exceeds 10 minutes."
The weekly physical activity will be noted in minutes.
Possible ranges: <150/ 150-300/ ≥300 perc
If the weekly physical activity is below 150 minutes.
YES
"Do you have any physical disability?"
Mark the answer with X. Options: yes/ no/ no data
If the answer is yes, than GUIDANCE 2
If the answer is no, than GUIDANCE 1
If the weekly physical activity ranges between 150-300 minutes, than POSITIVE FEEDBACK+ INCREASEA AMOUNT
If the weekly physical activity exceeds 300 minutes, than POSITIVE FEEDBACK

Opening sentences for every participant:

“The most common complication of the coronavirus infection are lung and heart disease, which has a major impact on the need for intensive care. If you perform enough physical activity, it can protect you from complications. Being in your home for long periods of time and because of this moving less can be a danger. We also advise you to avoid contact with others during physical activity as this may increase the risk of infection. ”

Guidance 1

“Your physical activity is less than the recommended 150 minutes per week. We recommend the following physical activities: leisure physical activity, commuting to work: walking or cycling, physical activity during work, housework, sports or planned physical activity. All activities should be performed in bouts of at least 10 minutes duration, with the recommended weekly duration being at least 3 * 50 or 5 * 30 minutes. If possible, stand up and move for 10 minutes after each hour spent sitting.”

Guidance 2

“Your physical activity is below the recommended 150 minutes per week, possibly increase it to over 150 minutes per week. For more detailed advice please contact your physician.”

Positive feedback + Increase amount

“Your regular physical activity reaches the recommended level, if possible, you can increase it to over 300 minutes a week. If possible, stand up and move for 10 minutes after each hour spent sitting.”

Positive feedback

“Your regular physical activity reaches the maximum recommended, and it is advisable to keep it at that level. If possible, stand up and move for 10 minutes after each hour spent sitting.”

First call
Nutrition and BMI

English protocol

Group B
Decision tree - 1st call

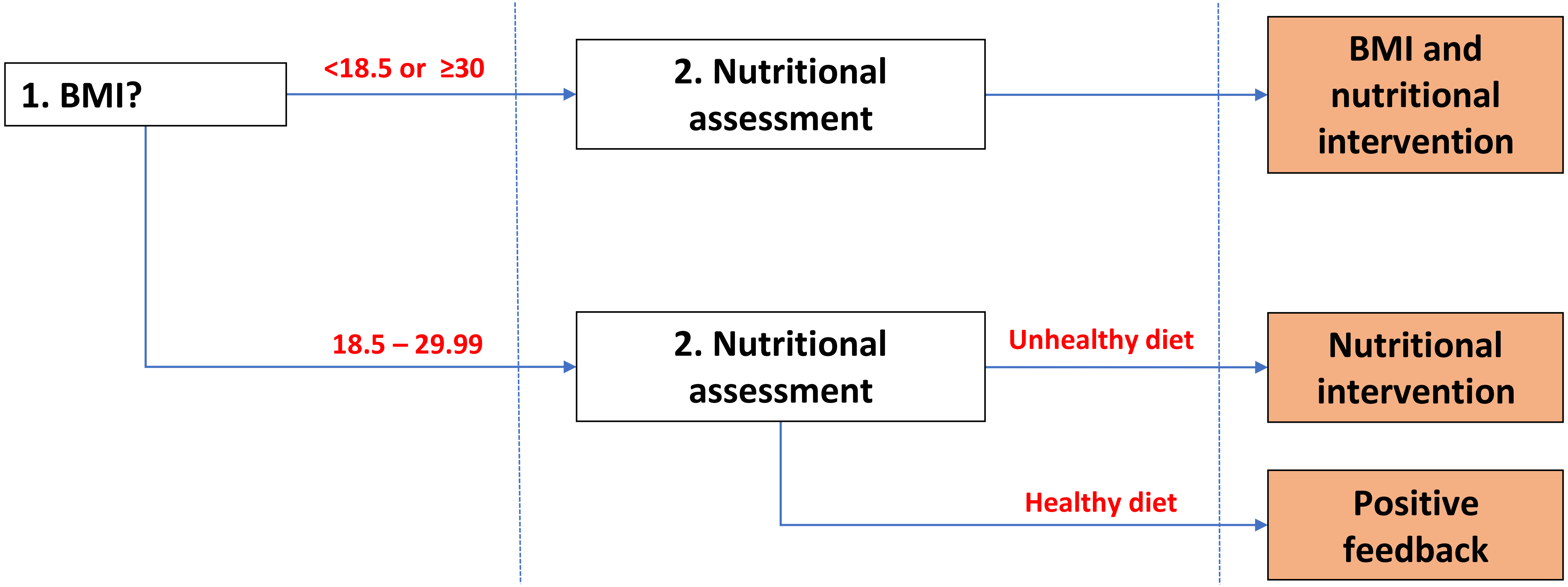

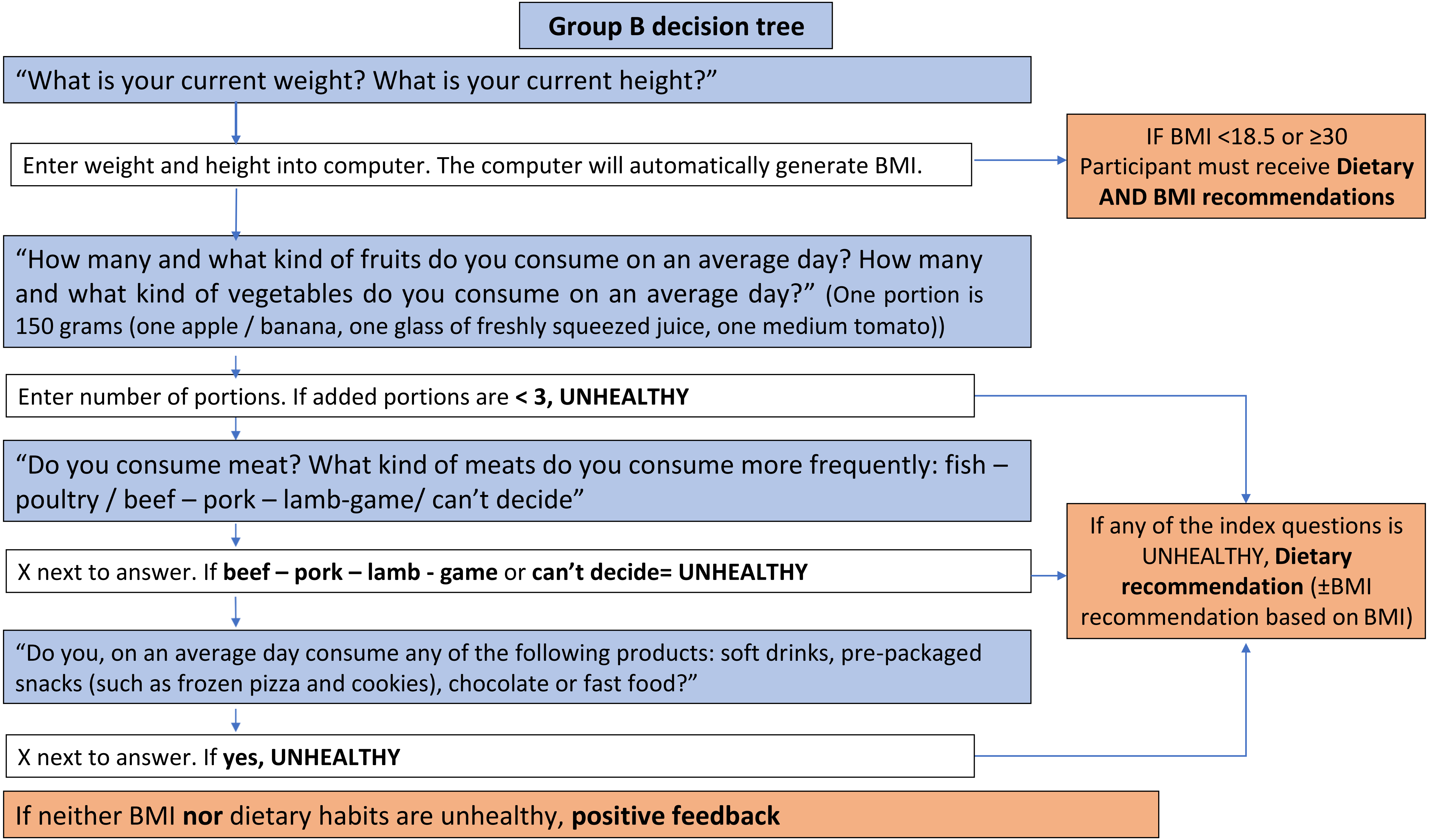
Group B decision tree
"What is your current weight? What is your current height?"
Enter weight and height into computer. The computer will automatically generate BMI.
IF BMI <18.5 or ≥30 Participant must receive **Dietary AND BMI recommendations**
"How many and what kind of fruits do you consume on an average day? How many and what kind of vegetables do you consume on an average day?" (One portion is 150 grams (one apple / banana, one glass of freshly squeezed juice, one medium tomato))
Enter number of portions. If added portions are **< 3, UNHEALTHY**
"Do you consume meat? What kind of meats do you consume more frequently: fish – poultry / beef – pork – lamb-game/ can't decide"
X next to answer. If **beef – pork – lamb - game** or **can't decide= UNHEALTHY**
If any of the index questions is UNHEALTHY, **Dietary recommendation** (±BMI recommendation based on BMI)
"Do you, on an average day consume any of the following products: soft drinks, pre-packaged snacks (such as frozen pizza and cookies), chocolate or fast food?"
X next to answer. If **yes, UNHEALTHY**
If neither BMI **nor** dietary habits are unhealthy, **positive feedback**

Beginning sentences for every participant

"We'd like to bring to your attention that an overly low or an overly high body weight poses an increased risk in terms of severity in every disease; thus, in the case of coronavirus, a more severe disease course is more likely to occur. Similarly, unhealthy dietary habits can negatively affect various factors including for example the risk of cardiovascular complications. With the current situation - leading to greater time being spent at home - the probability of gaining weight and taking to unhealthy dietary habits is higher."

Dietary recommendation:

(To the operator: The part where the index questions detected unhealthy consumption should be highlighted for the individuals.)

"We advise that you regularly consume fruits, vegetables, legumes (e.g., beans, peas, lentils), unsalted nuts and whole grain products (such as millet, oats, wheat and brown rice). You should consume at least 400 grams of vegetables and fruits every day (excluding potatoes!). Free sugars usually present in soft drinks and sweets are better avoided. Avoid consuming fatty foods, fat intake should amount for less than third of your daily calorie intake. Products containing unsaturated fats such as fish, nuts, sunflower- and olive oil are preferred, you should limit products containing saturated fats (such as fatty meat, cream and cheese) and you should avoid products containing trans-fats or additive such as some pastries and frozen foods, pre-packed snacks (crisps, cookies), fast-food items, cow meat."

"Naturally, if you are receiving dietary advice from a medical professional for health-related reasons, we recommend strict compliance with those recommendations."

BMI 18.5-29.99

"Compared to your body height, your body weight is ideal with regard to the risk of viral infection."

BMI recommendation

BMI <18.5: "Additionally, as your body weight is lower than the optimal range for your height, we recommend that you increase your consumption as possible."

BMI ≥30: "Additionally, as your body weight exceeds the optimal range for your height, we recommend that you decrease your consumption. It is important that this limitation is not extreme, because it could also pose a threat to your health and safety. We recommend 0.5-1 kg / week weight loss as a target."

Positive feedback

"Your dietary habits, as assessed by our questions, follow a healthy pattern. Still, we would like to inform you on the concepts of a healthy diet: that you regularly consume fruits, vegetables, legumes (e.g., beans, peas, lentils), unsalted nuts, and whole grain products (such as millet, oats, wheat and brown rice). You should consume at least 400 grams of vegetables and fruits every day (excluding potatoes!). Free sugars usually present in soft drinks and sweets are better avoided. Avoid consuming fatty foods, fat intake should amount for less than third of your daily calorie intake. Products containing unsaturated fats such as fish, nuts, sunflower- and olive oil are preferred, you should limit products containing saturated fats (such as fatty meat, cream and cheese) and you should avoid products containing trans-fats or additive such as some pastries and frozen foods, pre-packed snacks (crisps, cookies), fast-food items, cow meat."

First call
Alcohol consumption

English protocol

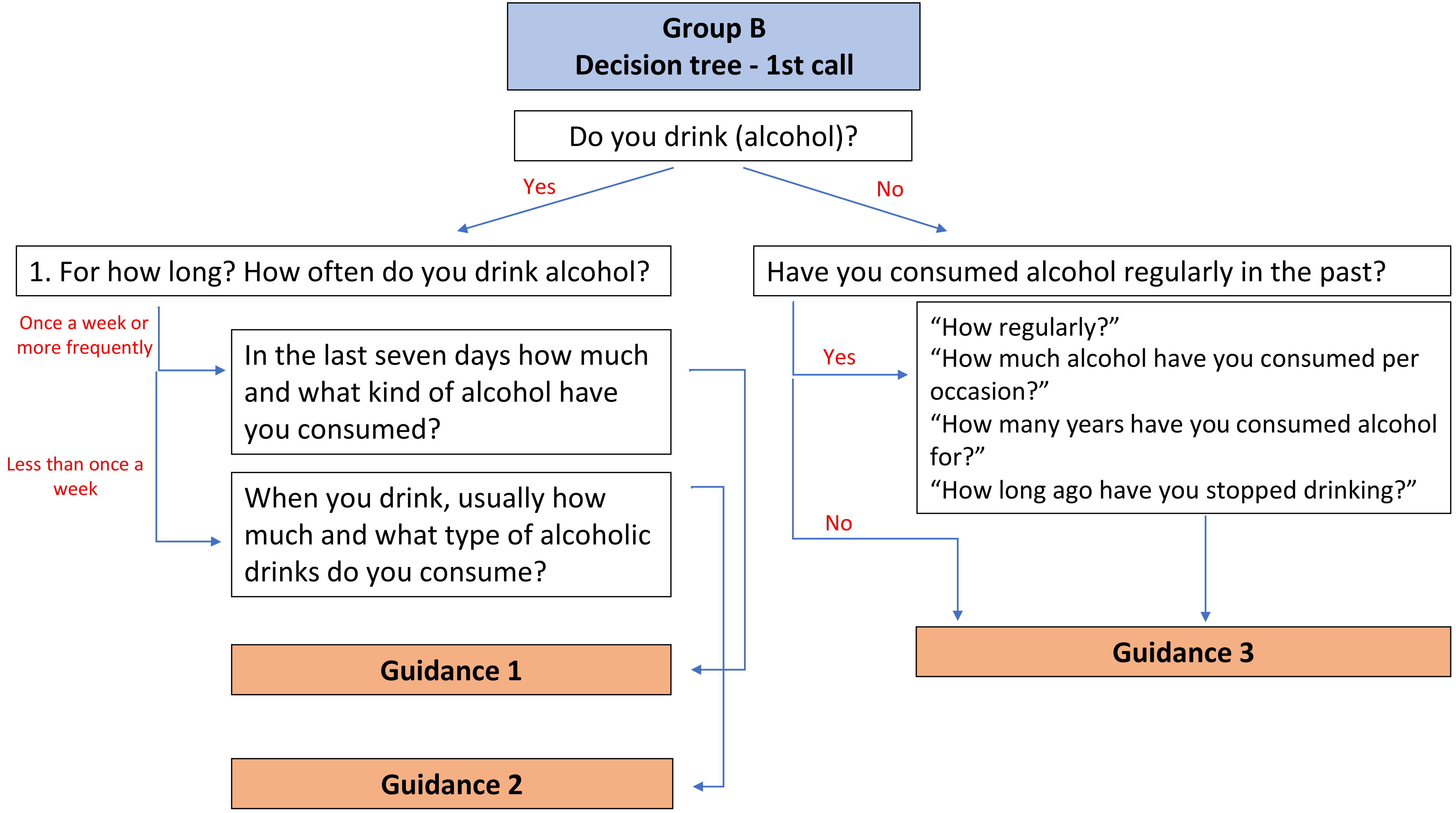
Group B
Decision tree - 1st call
Do you drink (alcohol)?
Yes
No
1. For how long? How often do you drink alcohol?
Once a week or more frequently
In the last seven days how much and what kind of alcohol have you consumed?
Less than once a week
When you drink, usually how much and what type of alcoholic drinks do you consume?
Guidance 1
Guidance 2
Have you consumed alcohol regularly in the past?
Yes
“How regularly?”
“How much alcohol have you consumed per occasion?”
“How many years have you consumed alcohol for?”
“How long ago have you stopped drinking?”
No
Guidance 3

Guidance 1: (Do not consume alcohol at all)

(To the operator: highlight what she/he consumes based on self-declaration).
“There are two very important things to know about alcohol consumption: Alcohol consumption is harmful, and it does not protect against the coronavirus.”
I advise you to consume as little alcohol as possible and do not exceed the following quantities:

- for men: 3 dL of wine or 3 glasses of beer or 6 cL hard drink
- for women: 2 dL of wine or 2 glasses of beer or 4 cL hard drink or 2 cocktails

Furthermore, do not consume more than 4 dL of wine or 4 glasses of beer or 8 cL of hard drink per occasion. The best thing you can do for your health is if you do not drink alcohol at all.”

Guidance 2: (Do not consume alcohol at all)

“There are two very important things to know about alcohol consumption: alcohol consumption is harmful, and it does not protect against the coronavirus. Keep in mind, that you should not consume more than 4 dL of wine or 4 glasses of beer or 8 cL of hard drink per occasion. The best thing you can do for your health is if you do not drink alcohol at all.”

Guidance 3: (Continue to consume no alcohol)

“You're doing great. Keep it up! Alcohol consumption has detrimental effects on health and does NOT protect you from coronavirus, we advise that keep alcohol-free!”

Repeated calls

Repeated calls
Stress

English protocol

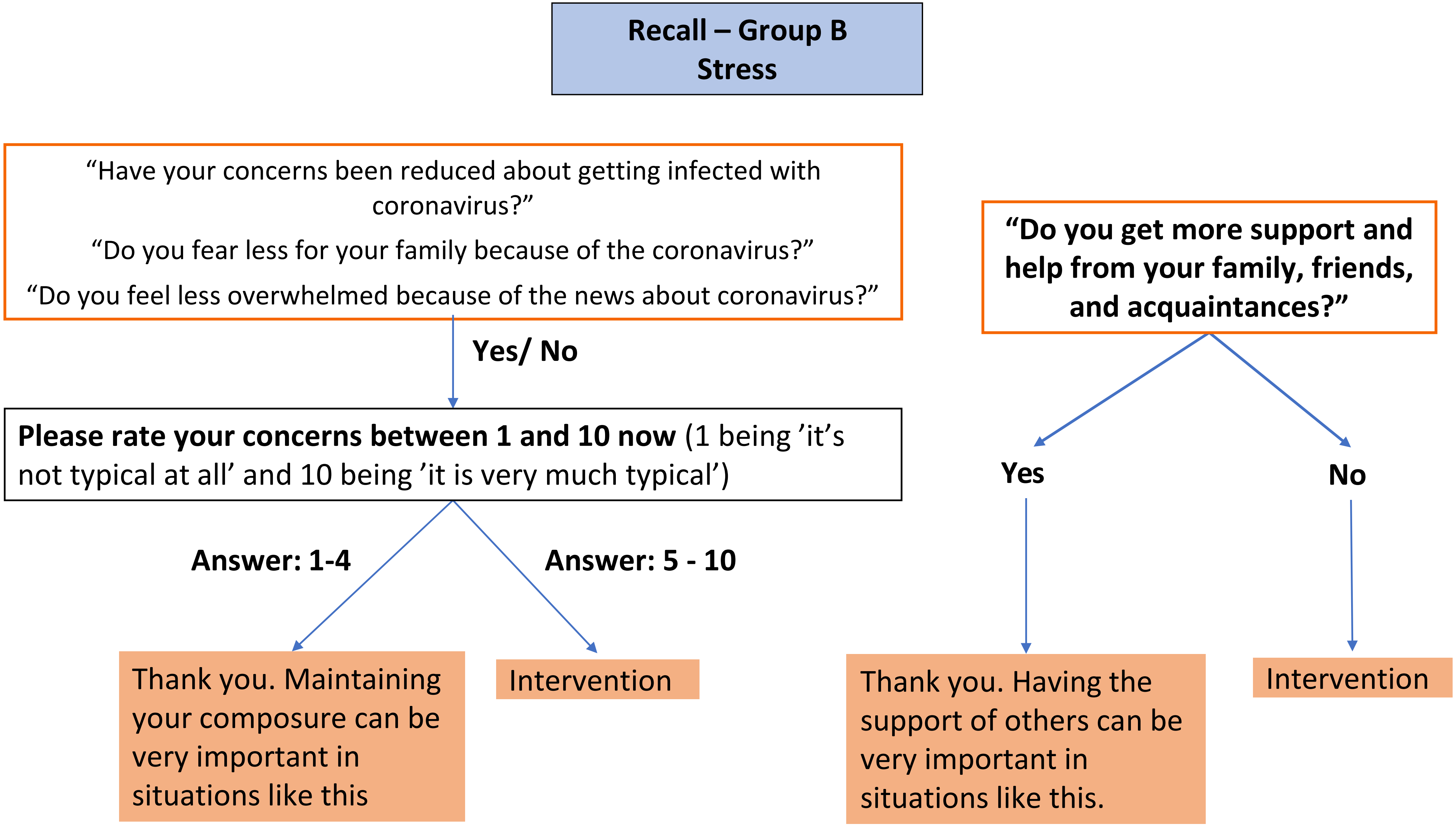
Recall – Group B
Stress
"Have your concerns been reduced about getting infected with coronavirus?"
"Do you fear less for your family because of the coronavirus?"
"Do you feel less overwhelmed because of the news about coronavirus?"
Yes/ No
Please rate your concerns between 1 and 10 now (1 being 'it's not typical at all' and 10 being 'it is very much typical')
Answer: 1-4
Answer: 5 - 10
Thank you. Maintaining your composure can be very important in situations like this
Intervention
"Do you get more support and help from your family, friends, and acquaintances?"
Yes
No
Thank you. Having the support of others can be very important in situations like this.
Intervention

Intervention

“As much as your opportunities allow, keep up your social connections, stay in touch with your family, friends, and acquaintances. If because of coronavirus related regulations you are required to avoid physical contact with others, try maintaining your social connections through other platforms, e.g. telephone, e-mail, social media (Facebook, TikTok, Skype, Twitter, etc.).

Establish a daily rhythm! Pay attention to your own needs and feelings. Engage in activities that you like and that make you feel calm, for example: watching movies or reading.

Share your thoughts with others! At the same time, you also should pay attention to the ones around you, listen to them, encourage them to share their experiences and feelings.

Constantly following news related to the epidemic can make you feel overwhelmed. Do not constantly follow this news, only periodically! Try to avoid all information that is not coming from a reliable source. You should primarily rely on news coming from healthcare professionals, for example the governmental information page: https://koronavirus.gov.hu/. Or dial any of the following informational lines: (06 80 277 455 / 06 80 277 456).

Because of the coronavirus related regulations, our social interactions can become limited easily, anybody can feel alone in a situation like this.

If you are feeling very alone, please let us know, and we can provide you a phone number that you can call to get professional help. (If the participant ask for the phone number: *Magyar Elsősegély Lelki Szolgálat*: 116-123.)”

Repeated calls
Smoking

English protocol

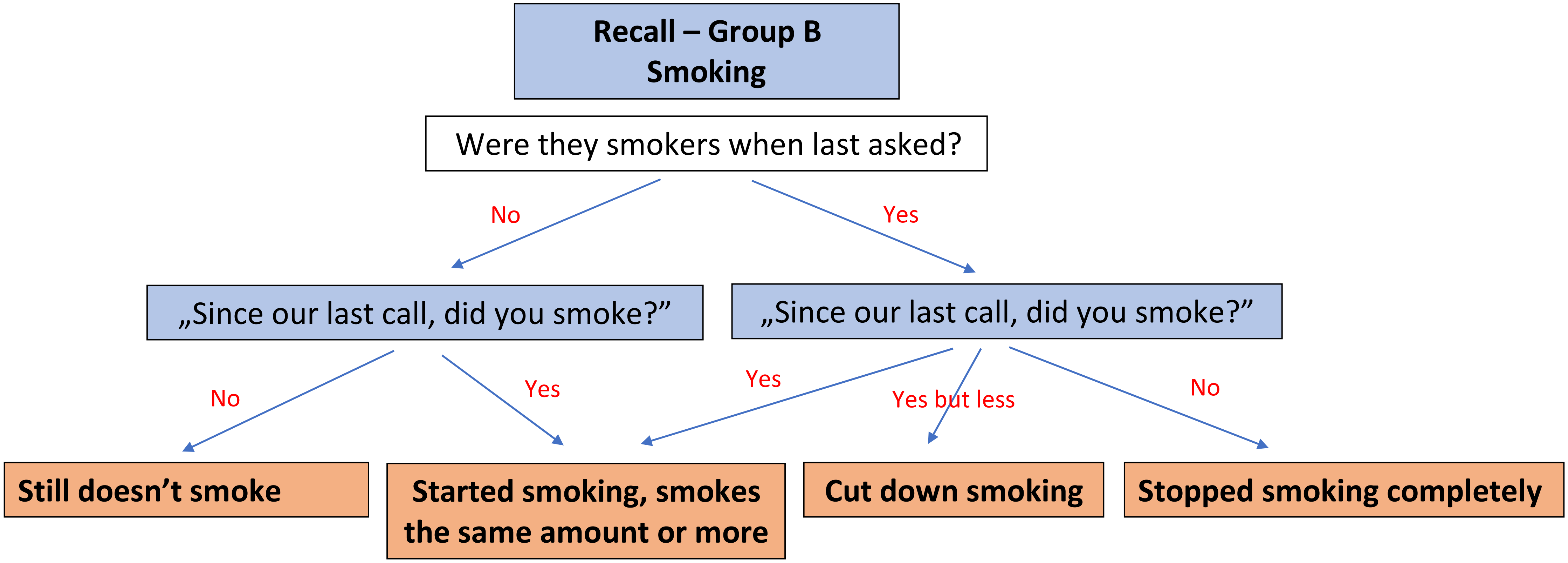
Recall – Group B
Smoking
Were they smokers when last asked?
No
Yes
„Since our last call, did you smoke?"
„Since our last call, did you smoke?"
No
Yes
Yes
Yes but less
No
Still doesn't smoke
Started smoking, smokes the same amount or more
Cut down smoking
Stopped smoking completely

Still doesn’t smoke

“Very good, keep it up and stay smoke-free. Upper airway infections are milder in non-smokers.”

Started smoking, smoking the same amount or increased that

“I see that you started smoking/ you are smoking the same amount/you increased the amount of smoking. Upper airway infections are milder in non-smokers. Accordingly, we can highly recommend that you stop smoking as soon as possible. If you are unable to do this, you might cut down smoking at least. If you need any further support to quit smoking, please let us know now.“
If yes: “To get help with quitting, call 06-80-44-20-44, which is a free service.”

Cut down smoking

“I see that you smoke less since our last call. You are doing well. Try to stop smoking completely. Upper airway infections are milder in non-smokers.”

Stopped smoking completely

“Congratulations, you have taken a very big step for your health since our last call. Keep it up, stay smoke-free. Upper airway infections are milder in non-smokers.”

Repeated calls
Physical activity

English protocol

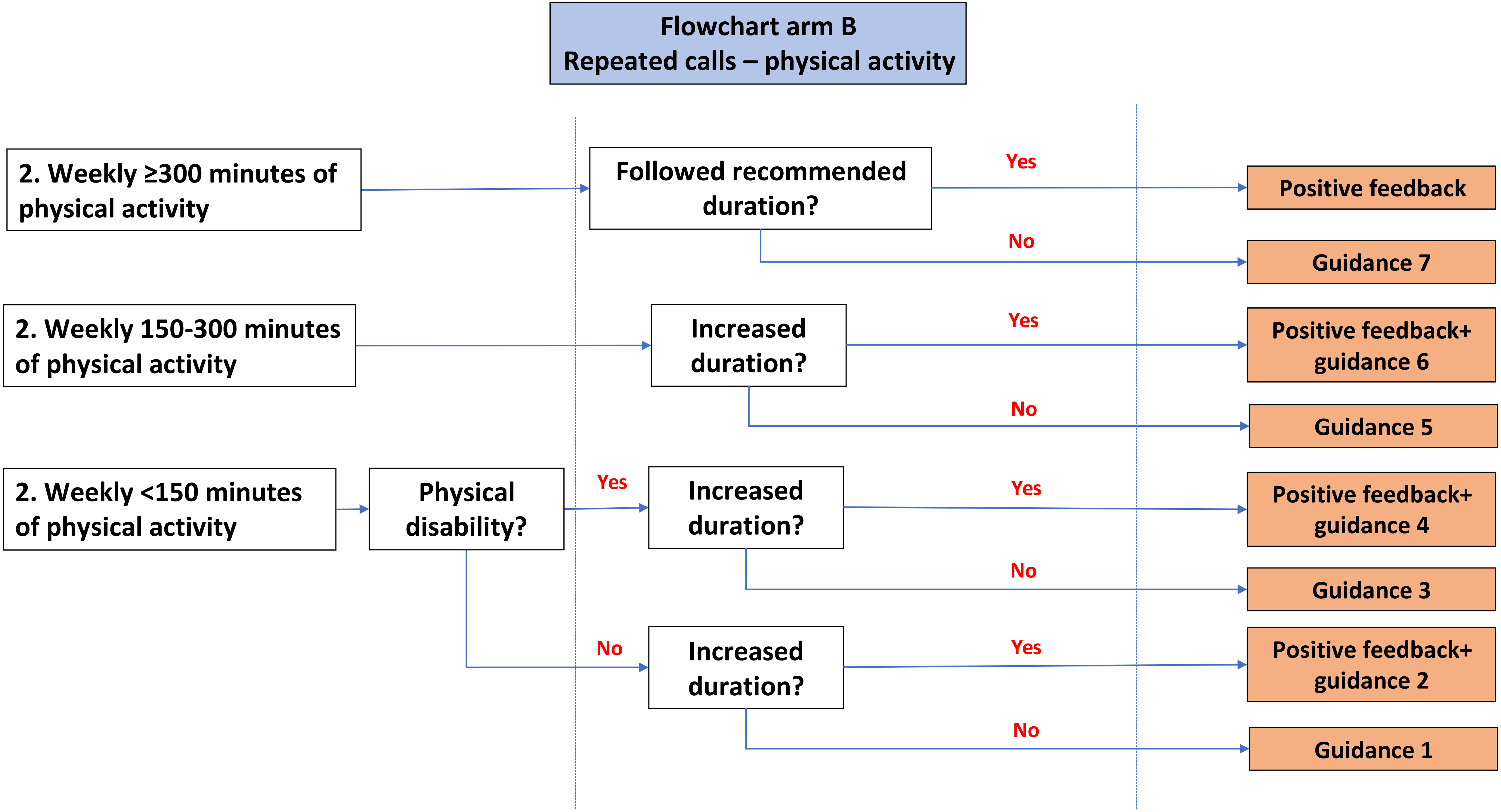
Flowchart arm B
Repeated calls – physical activity
2. Weekly ≥300 minutes of physical activity
Followed recommended duration?
Yes
Positive feedback
No
Guidance 7
2. Weekly 150-300 minutes of physical activity
Increased duration?
Yes
Positive feedback+ guidance 6
No
Guidance 5
2. Weekly <150 minutes of physical activity
Physical disability?
Yes
Increased duration?
Yes
Positive feedback+ guidance 4
No
Guidance 3
No
Increased duration?
Yes
Positive feedback+ guidance 2
No
Guidance 1

Opening

“The most common complications of the coronavirus infection are lung and heart disease, which has a major influence on the need for intensive care. If you perform enough physical activity, it can act as a protective factor against complications. Being home for long periods is a danger in terms of reduced physical activity. We recommend that you try to avoid any close contact with others during exercise because it can increase the risk of infection.”

Positive feedback

“You completed the 300 minutes of physical activity you have mentioned previously, therefore we recommend its continuation.”

Guidance 7

“You did not complete the 300 minutes of physical activity you have mentioned previously, so we recommend that you restore it.”

Positive feedback + Guidance 6

“You have successfully increased your weekly physical activity, you did great for your health. You can further increase this performance.”

Guidance 5

“You did not manage to increase your weekly physical activity. In order to do more for your health, you can further increase your performance."

Positive feedback + Guidance 4

"You have done a lot for your health, and we recommend that you continue with this."

Guidance 3

“In order to stay healthy, we recommend increasing your physical activity beyond 150 minutes per week. Contact your doctor for more detailed advice."

Positive feedback + Guidance 2

“You successfully increased your weekly 150 minutes of physical activity that you provided previously, you did a lot for your health. You can further increase this performance."

Guidance 1

“You did not increase your weekly 150 minutes of physical activity that you provided previously. In order to do more for your health, you can further increase this performance."

Repeated calls Nutrition and BMI

English protocol

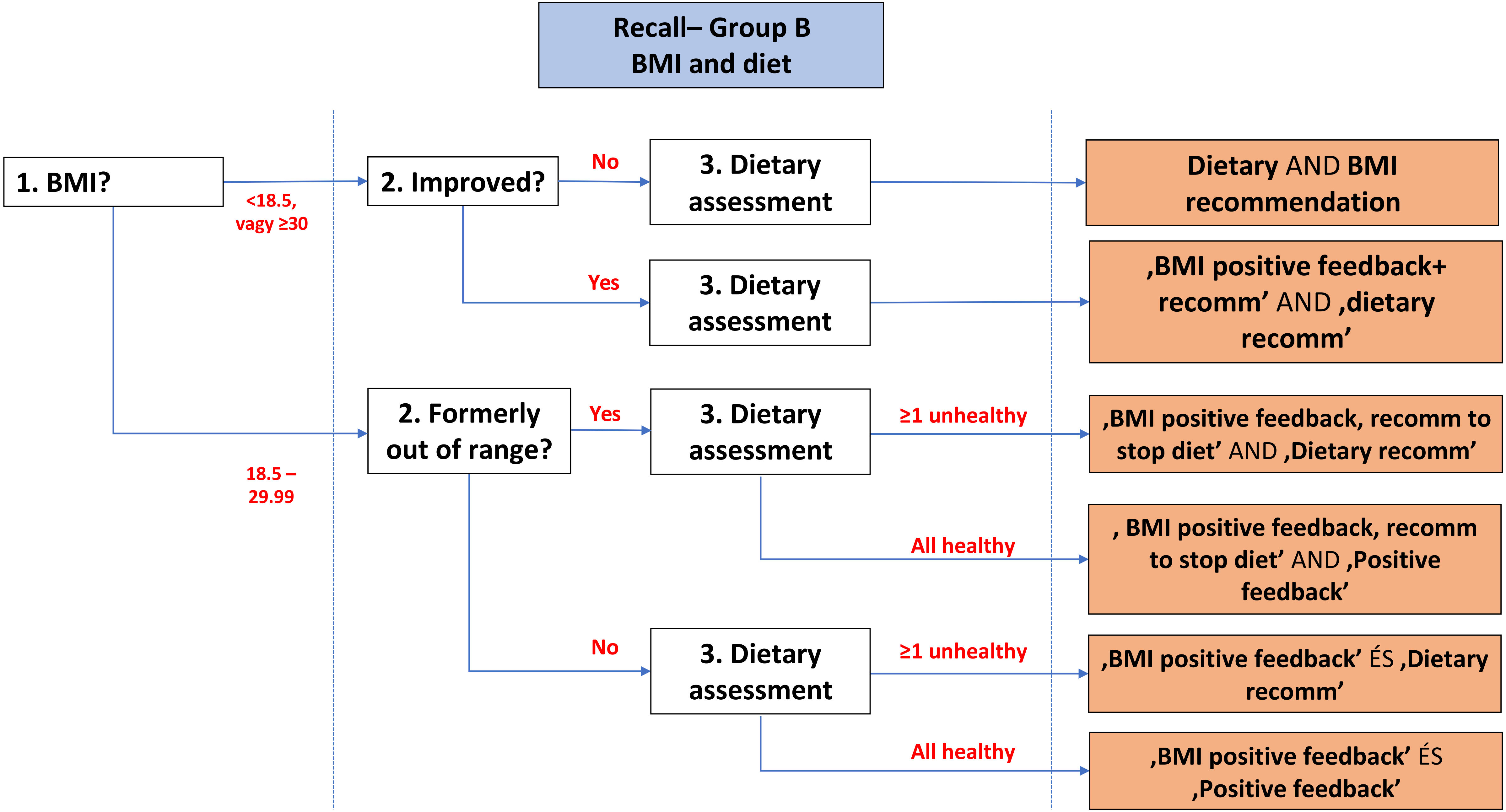

Recall– Group B
BMI and diet
1. BMI?
<18.5,
vagy ≥30
2. Improved?
No
3. Dietary assessment
Dietary AND BMI recommendation
Yes
3. Dietary assessment
‚BMI positive feedback+ recomm' AND ‚dietary recomm'
18.5 –
29.99
2. Formerly out of range?
Yes
3. Dietary assessment
≥1 unhealthy
‚BMI positive feedback, recomm to stop diet' AND ‚Dietary recomm'
All healthy
‚ BMI positive feedback, recomm to stop diet' AND ‚Positive feedback'
No
3. Dietary assessment
≥1 unhealthy
‚BMI positive feedback' ÉS ‚Dietary recomm'
All healthy
‚BMI positive feedback' ÉS ‚Positive feedback'

Opening lines:

“We’d like to bring to your attention to the fact that an overly low or an overly high body weight poses an increased risk in terms of severity in every disease; thus, it increases the risk of a more severe coronavirus infection. Similarly, unhealthy dietary habits can negatively affect various factors including, for example, the risk of cardiovascular complications. With the current situation - leading to greater time being spent at home - the probability of gaining weight and taking to unhealthy dietary habits is higher.”

Dietary recommendation:

The part where the index questions detected unhealthy consumption should be highlighted for the individuals.

“We advise that you regularly consume fruits, vegetables, legumes (e.g. beans, peas, lentils), unsalted nuts, and whole grain products (such as millet, oats, wheat and brown rice). You should consume at least 400 grams of vegetables and fruits every day (excluding potatoes!). Free sugars, usually present in soft drinks and sweets are better avoided. Avoid consuming fatty foods, fat intake should amount for less than a third of your daily calorie intake. Products containing unsaturated fats such as fish, nuts, sunflower- and olive oil are preferred, you should limit products containing saturated fats (such as fatty meat, cream, and cheese) and you should avoid products containing trans-fats and additives such any pastries and frozen foods, pre-packed snacks (crisps, cookies), fast-food items, meat of ruminant animals (such as cow).”
“Naturally, if you are receiving dietary advice from a medical professional for health-related reasons, we recommend strict compliance with those recommendations.”

BMI positive feedback:

“Compared to your body height, your body weight is ideal with regard to the risk of viral infection.”

BMI recommendations:

BMI <18.5: “Additionally, as your body weight is lower than the optimal range for your height, we recommend that you increase your food consumption as much as possible.”
BMI >30: “Additionally, as your body weight exceeds the optimal range for your height, we recommend that you decrease your consumption. It is important that this limitation must not be extreme, which could also pose a threat to your health and safety. We recommend 0.5-1 kg / week weight loss as a target.”

BMI positive feedback and recommendation:

“Based on the data you provided, your weight has improved since the last call, which is great news. As it still hasn’t reached the optimal range, we recommend that you continue losing/ gaining weight.”
+ dietary recommendation (the same)!

BMI positive feedback, recommendation to stop diet:

“Congratulations you’ve successfully lost/ gained weight, you reached the target optimal range. We do not recommend further weight loss/ gain.“

Positive feedback:

“Your dietary habits, as assessed by our questions, still follow a healthy pattern. Still, we would again like to inform you on the concepts of a healthy diet:
“We advise that you regularly consume fruits, vegetables, legumes (e.g. beans, peas, lentils), unsalted nuts, and whole grain products (such as millet, oats, wheat and brown rice). You should consume at least 400 grams of vegetables and fruits every day (excluding potatoes!). Free sugars, usually present in soft drinks and sweets are better avoided. Avoid consuming fatty foods, fat intake should amount for less than a third of your daily calorie intake. Products containing unsaturated fats such as fish, nuts, sunflower- and olive oil are preferred, you should limit products containing saturated fats (such as fatty meat, cream, and cheese) and you should avoid products containing trans-fats and additives such any pastries and frozen foods, pre-packed snacks (crisps, cookies), fast-food items, meat of ruminant animals (such as cow).”
“Naturally, if you are receiving dietary advice from a medical professional for health-related reasons, we recommend strict compliance with those recommendations.”

Repeated calls
Alcohol

English protocol

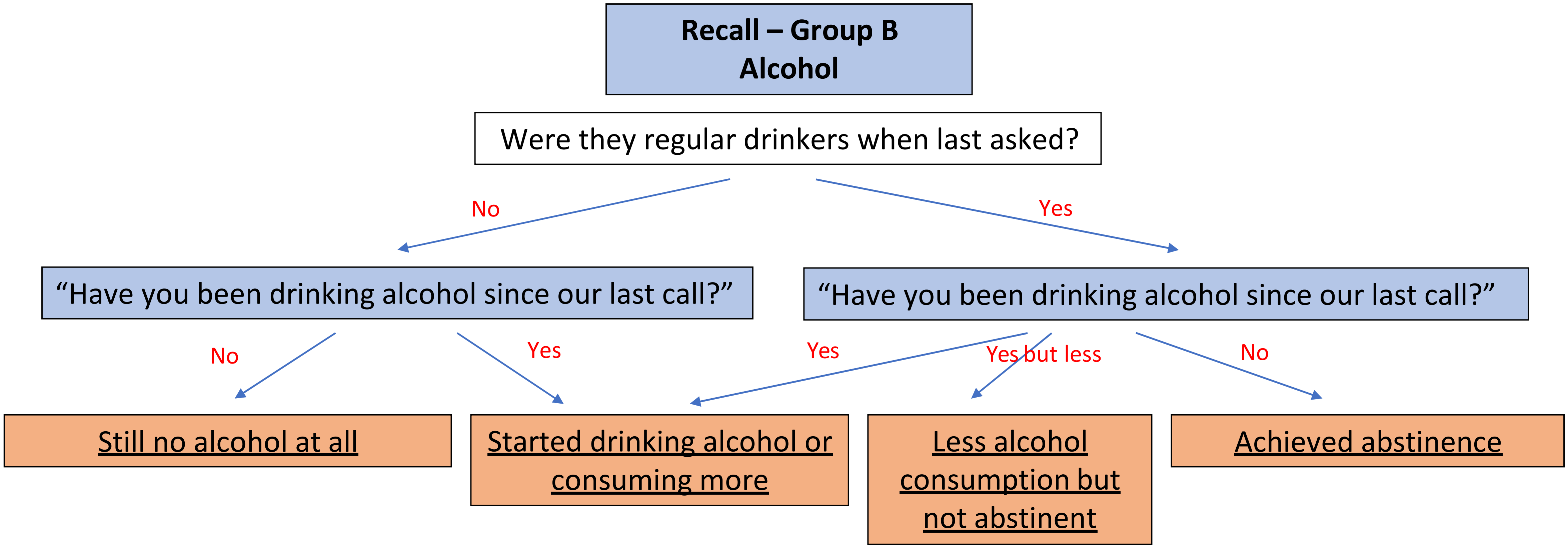
Recall – Group B
Alcohol
Were they regular drinkers when last asked?
No
Yes
"Have you been drinking alcohol since our last call?"
"Have you been drinking alcohol since our last call?"
No
Yes
Yes
Yes but less
No
Still no alcohol at all
Started drinking alcohol or consuming more
Less alcohol consumption but not abstinent
Achieved abstinence

Continued drinking no alcohol

"I see you still don't drink alcohol. You're doing great. Keep it up! Alcohol is harmful to the body and doesn't give protection against coronavirus infection."

Achieved abstinence

"Congratulations, you took a very big step in protecting your health. Maintain your abstinence! Alcohol is harmful to the body and doesn't give protection against coronavirus infection."

Less alcohol consumption but not abstinent

"We are glad that you drink less alcohol since the last conversation. You are on the right track! Try to stop drinking alcohol completely. Alcohol is harmful to the body and doesn't give protection against coronavirus infection."

Started drinking alcohol or consumes the same amount or more

(To the operator: highlight what she/he consumes based on self-declaration).

"I see that since our last phone call you started to drink alcohol/ drink more alcohol/ didn't change your alcohol consumption. There are two very important things to know about alcohol consumption: Alcohol consumption is harmful and it does not protect you against the coronavirus.

I advise you to consume as little alcohol as possible and do not exceed the following quantities:

- for men, 3 dL of wine, 3 glasses of beer, 6 cL hard drink (highlighting what he consumes based on self-declaration)
- for women 2 dL of wine, 2 glasses of beer, 4 cL hard drink, 2 cocktails

Furthermore, do not consume more than 4 dl of wine or 4 glasses of beer or 8 cL of hard drink per occasion. The best thing you can do for your health is if you do not drink alcohol at all."

Group A

All calls

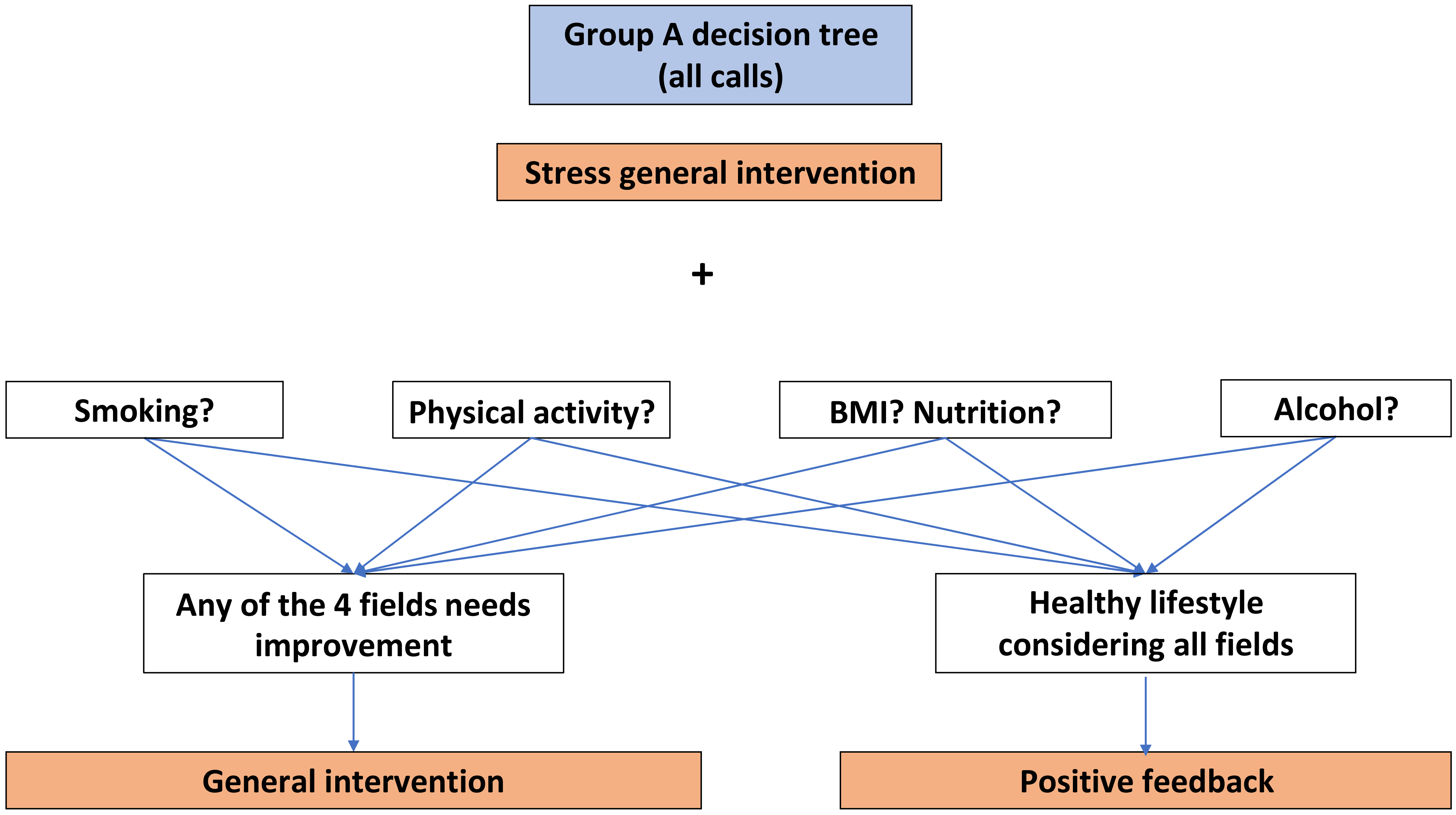
Group A decision tree
(all calls)
Stress general intervention
+
Smoking?
Physical activity?
BMI? Nutrition?
Alcohol?
Any of the 4 fields needs improvement
Healthy lifestyle considering all fields
General intervention
Positive feedback

Stress general intervention

"As far as possible, maintain interpersonal relationships, keep in touch with family friends or acquaintances.
Pay attention to your own needs and feelings. Engage yourself in activities that you enjoy and find calming.
Try to avoid any information coming from unreliable sources. Mainly rely on information coming from health professionals.
Because of the coronavirus related regulations, our social interactions can become limited easily, anybody can feel alone in a situation like this. If you are feeling very alone, please let us know, and we can provide you a phone number that you can call to get professional help. (If the participant ask for the phone number: *Magyar Elsősegély Lelki Szolgálat*: 116-123.)"

General intervention

"Smoking, physical activity, diet, and alcohol consumption influence the immune functions of your body. We recommend that - during the coronavirus pandemic - you try to avoid smoking and drinking alcohol, engage in regular physical activities for at least 150 minutes a week and eat healthy, because these factors may both prevent from getting infected with viruses and improve the severity of an infection. Alcohol is harmful for the body and doesn't give protection against coronavirus infection. The course of respiratory diseases is much milder in non-smokers. If you have any questions about these topics, please contact your family doctor for any further information."

Positive feedback

" Based on your answers, you are leading a healthy lifestyle. Considering that cessation of smoking and alcohol consumption, regular exercise, and a balanced diet can have a protective effect, we recommend that you continue to follow this lifestyle, because these factors may both prevent from getting infected with viruses and improve the severity of an infection. In addition, we would like to highlight the importance of drawing attention to these factors during public health and quarantine measures, for example, physical activity can be easily reduced and your weight easily increased. Alcohol is harmful to the body and doesn't give protection against coronavirus infection. The course of respiratory diseases is much milder in non-smokers. If you have questions about any of these topics, contact your physician."
