# Supplementary material for: Personalised health education against health damage of COVID-19 epidemic in the elderly Hungarian population (PROACTIVE-19): protocol of an adaptive randomised controlled clinical trial
Source: Trials. 2020 Sep 29;21:809. doi: 10.1186/s13063-020-04733-0 (PMC7522906; doi:10.1186/s13063-020-04733-0)
Supplement: Supplementary file 3 — Additional file 3. [file 13063_2020_4733_MOESM3_ESM.pdf]

PROACTIVE-19 QUESTIONS AND INTERVENTIONS

The study participants will be randomized into two groups (group A and B). Group A will receive general guidance about the importance of healthy lifestyle. Group B will receive personalized advice about the lifestyle changes that are necessary to improve their health status. Later on, data recording and intervention will be repeated in both groups on a weekly basis in the first month, every two weeks in the second month, and monthly from the third month.

Interventions of the PROACTIVE-19 study are detailed below.

The structure of the call in Group A:
1. interview of the participants (mental stress, smoking, physical activity, diet, alcohol consumption)
2. general counselling

The structure of the call in Group B:
1. mental stress (interview followed by personalized counselling)
2. smoking (interview followed by personalized counselling)
3. physical activity (interview followed by personalized counselling)
4. diet (interview followed by personalized counselling)
5. alcohol consumption (interview followed by personalized counselling)

1st CALL

“Good morning / afternoon I’m XY call center coordinator for the PROACTIVE-19 study. Excuse us for the long information, it was necessary to meet legal requirements. First, I have to ask, that...”

"Has anyone in your household already called this number?"

If yes: **"Follow the recommendations given to your relative. If your lifestyle is significantly different, please mention it now.”**

(To the operator: depending on the information what the caller provides, give advice in the appropriate category).

NO DATA COLLECTION, NO RANDOMIZATION, NO FURTHER TELEPHONE CALL.

“Have you ever been diagnosed with coronavirus infection?”

If yes: **“Thank you for your interest, but in this trial we can only enroll people who have not been infected with coronavirus. Our best wishes attend you, goodbye!”**

If no: **„Please repeat after me the following statement.”**

“I – XXX name, place and date of birth - ‘at current date XXX’, after I have been informed by Dr. Péter Hegyi principal investigator and XXX (name of the operator), and I have had my questions regarding this answered to my satisfaction. I give my consent to participate in this study of the University of Pécs voluntarily, without influence, after I have received detailed information. I am aware that I may withdraw my consent at any time, verbally or in writing, without explanation.”

“Thank you”

“First, I’d like to ask for some personal data, related to the attendance.

“Your current name?”

“Your birth name?”

“Place of birth?”

“Postal code? “

“Telephone number? “ **“Date of birth?”**

“Insurance number?”

RANDOMISATION

GROUP A

QUESTIONS

(Questions are to be completed in an identical manner in both groups)

"Okay. "In the following minutes, I would like to ask some questions in order to assess your current lifestyle, then you will receive recommendations according to this. These will include 5 domains: stress, smoking, physical activity, dietary habits, and alcohol consumption. It is very important that you try and give accurate answers as this would allow us to give you the best recommendations. All information will be handled in an anonymous manner. Please have a pen and a paper next to you as you might have to write some information down."

"First I'd like to ask some questions related to your worries."

"If you'd have to choose a number between 1 and 10, 1 being 'it's not typical at all' and 10 being 'very much typical'... "

"How much do you worry about getting infected with coronavirus?"

"How much do you fear for your family because of coronavirus?"

"How overwhelmed do you feel because of the news about coronavirus?"

"Finally, I would like to ask…"

"Do you receive adequate support and help from your friends, family, or acquaintances?"

All right, thank you. Now, I would like to ask you about your smoking habits.
Do you currently smoke?
- If yes:
- **Do you smoke regular cigarettes?**
  - If yes:
  - **For how many years have you been smoking?**
  - **How many cigarettes do you smoke per day?**
- **Do you use electronic cigarettes?**
- If no:
- **Have you ever smoked in the past?**
  - if yes:
  - **How long ago have you stopped smoking?**
  - **Altogether, for how many years did you smoke for?**
  - **Back then, how many cigarettes did you smoke per day?**

"In the following, we would like to ask you about your physical activity."

"How much time do you spend with physical activity weekly? By this, we mean gardening, housework, or any physical activity which is longer than 10 minutes."

(To the operator: this data should be noted in minutes; if the study participant responds in hours, it should be transformed to minutes.)

(To the operator: the definition of physical activity is important; the minimum duration of physical activity should be at least 10 minutes, and includes the following activities: leisure physical activity, physical activity at work, housework, sport, or planned physical activity.)

If the weekly physical activity is less than 150 minutes:

"Do you have any physical disabilities?"

(To the operator: the possible answers are yes or no. Physical disability includes: bedridden, wheelchair bound, limb amputated, stroke patients.)

"Next I'd like to ask about your <u>weight and dietary</u> habits."
"What is your current weight?
"What is your current height?"
"How many and what kind of fruits do you consume on an average day?"
(To the operator: one portion approximately equals one piece of a bigger fruit (eg. apples, bananas, oranges) or alternatively one glass of freshly squeezed juice.)
"How many and what kind of vegetables do you consume on an average day?"
(To the operator: one portion approximately equals a medium tomato or a bigger pepper. Potato consumption doesn't count!)
(To the operator: one portion in the case of fruits / vegetables: 150 grams)
"Do you consume meat?

If yes:
"What kind of meats do you consume more frequently: fish – poultry / beef – pork – lamb-game / can't pick from the two"

"Do you, on an average day consume any of the following products: soft drinks, pre-packaged snacks (such as frozen pizza and cookies), chocolate or fast food?"

"Thank you. Lastly, I would like to ask about your <u>drinking</u> habits. Do you drink alcohol?"

If yes:
"**„For how long?**"
"How often do you drink alcohol?"

If once a week or more frequently:
"In the last seven days, how much and what kind of alcohol have you consumed?"
If less than once a week:
„When you drink, usually how much and what type of alcoholic drinks do you consume?"

If no:
"Have you consumed alcohol regularly in the past?"

If yes:
"How frequently?"
"How much alcohol have you consumed per occasion?"
"For how many years have you consumed alcohol?"
"How long ago have you stopped drinking?"

GENERAL INTERVENTION

STRESS GENERAL INTERVENTION

As far as possible, maintain interpersonal relationships, keep in touch with family friends, and acquaintances.

Pay attention to your own needs and feelings. Engage yourself in activities that you enjoy and find calming.

Try to avoid any information coming from unreliable sources. Mainly rely on information coming from health professionals.

Because of the coronavirus related regulations, our social interactions can become limited easily, anybody can feel alone in a situation like this.

During an epidemic, social isolation can easily occur, leading to the feeling of loneliness. If you are feeling very alone and you'd like to receive help please say so and we can provide you with contact information for professional help."

If yes: *Magyar Elsősegély Lelki Szolgálat*: 116-123.

If the lifestyle is unhealthy in any aspect: Smoking, physical activity, diet, and alcohol consumption influence the immune functions of your body. We recommend that - during the coronavirus pandemic - you try to avoid smoking and drinking alcohol, engage in regular physical activities for at least 150 minutes a week and eat healthy, because these factors may both prevent from getting infected with viruses and improve the severity of an infection. Alcohol is harmful for the body and doesn't give protection against coronavirus infection. The course of respiratory diseases is milder in non-smokers. If you have any questions about these topics, please contact your family doctor for any further information.

Positive feedback: Based on your answers, you are leading a healthy lifestyle. Considering that cessation of smoking and alcohol consumption, regular exercise, and a balanced diet can have a protective effect, we recommend that you continue to follow this lifestyle, because these factors may both prevent from getting infected with viruses and improve the severity of an infection. In addition, we would like to highlight the importance of drawing attention to these factors during public health and quarantine measures, for example, physical activity can be easily reduced and your weight easily increased. Alcohol is harmful for the body and doesn't give protection against coronavirus infection. The course of respiratory diseases is much milder in non-smokers. If you have questions about any of these topics, contact your physician.

Thank you for your participation! We hope that we could help you with our advice. For a regular, not just an occasional health improvement, we will call you again in 1 week / 2 weeks / a month. Next time you won't have to listen to the long information at the beginning of the phone call again. Please don't put down the phone yet, because we would like to ask you to give us a really short feedback with the help of the buttons.

Goodbye!

GROUP B

(Questions are to be completed in an identical manner in both groups)

“Okay. Next we will ask questions relating to your lifestyle. We will cover 5 domains: stress, smoking, physical activity, dietary habits and alcohol consumption. It is very important that you try and give accurate answers as this would allow us to give you the best recommendations. All information will be handled in an anonymous manner. Please have a pen and a paper next to you as you might have to write some information down.”

“First I’d like to ask some questions relating to your worries.”

“If you’d have to choose a number between 1 and 10, 1 being ’it’s not typical at all’ and 10 being ’it is completely typical’... ”

1. “How much do you worry about getting infected with coronavirus?”

2. “How much do you fear for your family because of coronavirus”

3. “How overwhelmed do you feel because of the news about coronavirus?”

“Finally, I would like to ask…”

4. “Do you receive proper support and help from your friends, family or acquaintances?”

STRESS PERSONAL INTERVENTION

If questions 1-3 are on average >4 OR question 4 is NO:
As much as your opportunities allow you to keep up your social connections, stay in touch with your family, friends, and acquaintances. If because of coronavirus related regulations you are required to avoid physical contact with others, try maintaining your social connections through other platforms, e.g. telephone, e-mail, social media (Facebook, TikTok, Skype, Twitter, etc.).

Establish a daily rhythm! Pay attention to your own needs and feelings. Engage in activities that you like and that make you feel calm, for example: watching movies or reading.

Share your thoughts with others! At the same time, you also should pay attention to the ones around you, listen to them, encourage them to share their experiences and feelings.

Constantly following news related to the epidemic can make you feel overwhelmed. Try to follow these news only periodically! Try to avoid all information that is not coming from a reliable source. You should primarily rely on news coming from healthcare professionals, for example the governmental information page: https://koronavirus.gov.hu/. Or dial any of the following informational lines: (06 80 277 455 / 06 80 277 456).
Because of the coronavirus related regulations, our social interactions can become limited easily, anybody can feel alone in a situation like this.
If you are feeling really alone, you should seek professional help. For example, call the *’Magyar Lelki elsősegély* telefonszolgálat’: 116-123

If questions 1-3 are on average <=4 and question 4 is YES:

Thank you. Maintaining your composure can be very important in situations like this. Having the support of others can help a lot.

SMOKING QUESTIONS

"Now, I would like to ask you about your smoking habits."
"Do you currently smoke?"
If yes:
" Do you smoke regular cigarettes?"
If yes: "**How many years have you been smoking for?"**
"How many cigarettes do you smoke per day?"
"**Do you use electronic cigarettes?"**
If no:
"Have you ever smoked in the past?"
if yes:
"How long ago have you stopped smoking?"
"Altogether, how many years did you smoke for?"
"Back then, how many cigarettes did you smoke per day?"

SMOKING PERSONALIZED INTERVENTION

We recommend you to stop smoking: "To our knowledge, among the lifestyle factors, tobacco smoking has the greatest impact on the outcome of coronavirus cases. The course of the disease is milder in non-smokers. Accordingly, we can highly recommend that you stop smoking as soon as possible. If you are unable to do this, you might cut down smoking at least. If you need any further support to quit smoking, please let us know now."
If yes: "To get help with quitting, call 06-80-44-20-44, which is a free service."

We recommend you to stay smoke-free: „Very good, keep it up, and stay smoke-free! The course of the disease is much milder in non-smokers.

PHYSICAL ACTIVITY QUESTIONS
In the following, we would like to ask you about your physical activity. Please answer yes or no:**"How much time do you spend with physical activity weekly? By this, we mean gardening, housework or any physical activity which is longer than 10 minutes."**

(To the operator: this data should be noted in minutes; if the study participant responds in hours, it should be transformed to minutes.)

(To the operator: the definition of physical activity is important; the minimum duration of physical activity should be at least 10 minutes, and includes the following activities: leisure physical activity, physical activity at work, housework, sport or planned physical activity.)

If the weekly physical activity is less than 150 minutes:

"Do you have any physical disabilities?"

(To the operator: the possible answers are yes or no. Physical disability includes: bedridden, wheelchair bound, limb amputated, stroke patients.)

PHYSICAL ACTIVITY PERSONALIZED INTERVENTION

(In every case, counselling starts with the opening sentence, then it continues according to the decision tree.)

Opening with: "The most common complications of the infection are lung and heart disease, which has a major influence on the need for intensive care. If you perform adequate amounts of physical activity, it can act as a protective factor against complications. Being home for long periods of time is a riskin terms of reduced physical activity. We recommend that you try to avoid any close contact with others during exercise, because it can increase the risk of infection."

Positive feedback (if the weekly physical activity is more than 300 minutes):

"Your regular physical activity reaches the maximum recommended level, and it is advisable to keep it at that level. If possible, stand up and move for 10 minutes after each hour spent sitting."

Positive feedback and increase amount (if the weekly physical activity is between 150-300 minutes):

"Your regular physical activity reaches the recommended level, if possible, you can gradually increase it to over 300 minutes a week, because it has further advantages for your health. If possible, stand up and move for 10 minutes after each hour spent sitting."

Guidance 1 (if the weekly physical activity is less than 150 minutes):

"Your physical activity is less than the recommended 150 minutes per week. We recommend the following physical activities: leisure physical activity, physical activity during work, housework, sports, or planned physical activity. All activities should be performed in bouts of at least 10 minutes duration, with the recommended weekly duration being at least 3 * 50 or 5 * 30 minutes. If possible, stand up and move for 10 minutes after each hour spent sitting."

Guidance 2 (if the weekly physical activity is less than 150 minutes and physical disability is present):
"Your physical activity is below the recommended 150 minutes per week, possibly increase it to over 150 minutes per week. For more detailed advice please contact your physician."

BMI AND DIETARY QUESTIONS
"Next I'd like to ask about your weight and dietary habits."
"What is your current weight?
"What is your current height?"
"How many and what kind of fruits do you consume on an average day?"
(To the operator: one portion approximately equals one piece of a bigger fruit (eg. apples, bananas, oranges) or alternatively one glass of freshly squeezed juice.)
"How many and what kind of vegetables do you consume on an average day?"
(To the operator: one portion approximately equals a medium tomato or a bigger pepper. Potato consumption doesn't count!)
(To the operator: one portion in the case of fruits / vegetables: 150 grams)
"Do you consume meat?

If yes:
"What kind of meats do you consume more frequently: fish – poultry / beef – pork – lamb-game/ can't decide."

“Do you, on an average day consume any of the following products: soft drinks, pre-packaged snacks (such as frozen pizza and cookies), chocolate or fast food?”

BMI AND DIETARY PERSONALIZED INTERVENTION

Beginning sentences for every participant: „We’d like to bring to your attention that an overly low or an overly high body weight poses an increased risk in terms of severity in every disease; thus, in the case of coronavirus, a more severe disease course is more likely to occur. Similarly, unhealthy dietary habits can negatively affect various factors including for example the risk of cardiovascular complications. With the current situation - leading to greater time being spent at home - the probability of gaining weight and taking to unhealthy dietary habits is higher.”

BMI 18.5-29.99: “Compared to your body height, your body weight is ideal regarding the risk accompanying the viral infection.”

BMI <18.5: „Additionally, as your body weight is lower than the optimal range for your height, we recommend that you increase your consumption as possible.”

BMI >30: „Additionally, as your body weight exceeds the optimal range for your height, we recommend that you decrease your consumption. It is important that this limitation is not extreme, because it could also pose a threat to your health and safety. We recommend 0.5-1 kg / week weight loss as a target.”

Dietary recommendation:
(To the operator: The part where the index questions detected unhealthy consumption should be highlighted for the individuals.)
„We advise that you regularly consume fruits, vegetables, legumes (e.g., beans, peas, lentils), unsalted nuts and whole grain products (such as millet, oats, wheat and brown rice). You should consume at least 400 grams of vegetables and fruits every day (excluding potatoes!). Free sugars usually present in soft drinks and sweets are better avoided. Avoid consuming fatty foods, fat intake should amount for less than third of your daily calorie intake. Products containing unsaturated fats such as fish, nuts, sunflower- and olive oil are preferred, you should limit products containing saturated fats (such as fatty meat, cream and cheese) and you should avoid products containing trans-fats or additive such as some pastries and frozen foods, pre-packed snacks (crisps, cookies), fast-food items, cow meat.”

„Naturally, if you are receiving dietary advice from a medical professional for health-related reasons, we recommend strict compliance with those recommendations.”

Positive feedback: “Your dietary habits, as assessed by our questions, follow a healthy pattern. Still, we would like to inform you on the concepts of a healthy diet: that you regularly consume fruits, vegetables, legumes (e.g., beans, peas, lentils), unsalted nuts, and whole grain products (such as millet, oats, wheat and brown rice). You should consume at least 400 grams of vegetables and fruits every day (excluding potatoes!). Free sugars usually present in soft drinks and sweets are better avoided. Avoid consuming fatty foods, fat intake should amount for less than third of your daily calorie intake. Products containing unsaturated fats such as fish, nuts, sunflower- and olive oil are preferred, you should limit products containing saturated fats (such as fatty meat, cream and cheese) and you should avoid products containing trans-fats or additive such as some pastries and frozen foods, pre-packed snacks (crisps, cookies), fast-food items, cow meat.”

„Naturally, if you are receiving dietary advice from a medical professional for health-related reasons, we recommend strict compliance with those recommendations.”

ALCOHOL CONSUMPTION QUESTIONS

“Thank you. Lastly, I would like to ask about your drinking habits. Do you drink alcohol?"

If yes:

“**For how long?**”

“**How often do you drink alcohol?**”

If once a week or more frequently:

"**In the last seven days, how much and what kind of alcohol have you consumed?**"

If less than once a week:

”When you drink, usually how much and what type of alcoholic drinks do you consume?”

If no:

“Have you consumed alcohol regularly in the past?”

If yes:

“How regularly?”

“How much alcohol have you consumed per occasion?”

“How many years have you consumed alcohol for?”

“How long ago have you stopped drinking?”

ALCOHOL CONSUMPTION PERSONALIZED INTERVENTION

(To the operator: highlight what she/he consumes based on self-declaration).

Guidance 1 (do not consume alcohol at all): „There are two very important things to know about alcohol consumption: Alcohol consumption is harmful, and it does not protect against the coronavirus.”
I advise you to consume as little alcohol as possible and do not exceed the following quantities:
- for men: 3 dL of wine or3 glasses of beer or6 cL hard drink
- for women: 2 dL of wine or2 glasses of beer or4 cL hard drink or2 cocktails

Furthermore, do not consume more than 4 dL of wine or 4 glasses of beer or 8 cL of hard drink per occasion. The best thing you can do for your health is if you do not drink alcohol at all.”

Guidance 2 (do not consume alcohol at all): „There are two very important things to know about alcohol consumption: alcohol consumption is harmful, and it does not protect against the coronavirus. Keep in mind, that you should not consume more than 4 dL of wine or 4 glasses of beer or 8 cL of hard drink per occasion. The best thing you can do for your health is if you do not drink alcohol at all.”
Guidance 3 (continue to consume no alcohol): “You're doing great. Keep it up! Alcohol consumption has detrimental effects on health and does NOT protect you from coronavirus, we advise that keep alcohol-free!

Thank you for your participation! We hope that we could help you with our advice. For a regular, not just an occasional health improvement, we will call you again in 1 week / 2 weeks / a month. Next time you won’t have to listen to the long information at the beginning of the phone call again. Please don’t put down the phone yet, because we would like to ask you to give us a really short feedback with the help of the buttons.
Goodbye!
