# Supplementary material for: Personalised health education against health damage of COVID-19 epidemic in the elderly Hungarian population (PROACTIVE-19): protocol of an adaptive randomised controlled clinical trial
Source: Trials. 2020 Sep 29;21:809. doi: 10.1186/s13063-020-04733-0 (PMC7522906; doi:10.1186/s13063-020-04733-0)
Supplement: Supplementary file 4 — Additional file 4. [file 13063_2020_4733_MOESM4_ESM.pdf]

RECALL (Second and later calls)

(Questions are to be completed in an identical manner in both groups!)

If a relative picks up the phone:
Says the participant is dead: need to be recorded, no further calls
Says that the participant is in the hospital:
"Because of the coronavirus or some other reason?"
"Date of hospitalization............ "

If the participant picks up the phone:
"Good morning/ afternoon I'm XY call center coordinator for the PROACTIVE-19 study. Last time we asked a series of questions about your lifestyle then gave recommendations. This conversation will follow a similar structure.
Like last time it is very important that you try and give accurate answers as this would allow us to give you the best recommendations. All information will be handled in an anonymous manner. These will include the same 5 domains: your worries, smoking, physical activity, dietary habit and alcohol consumption."

"First, I'd like to ask some general questions."
"**Have you had a coronavirus test lately?"** (yes/no/no data)
If yes: **"What was the test result?"** (positive/negative/in progress/do not know)
"Since the last call, have you been admitted to the hospital because of a coronavirus infection?" (yes/no/no data)
If yes: "If you have been infected with coronavirus, this trial is over for you. Of course, if you take the advice about healthy lifestyle, you can further improve your health status. Our best wishes attend you, thank you for your participation! Goodbye!"
"**Are you in hospital right now because of coronavirus infection?"** (yes/no/no data)
If yes: "In this case, we are hoping for your speedy recovery. We won't call you because of this trial anymore. With the help of life-style advice you can improve your health further. Our best wishes attend you, thank you for your participation! Goodbye!"

Participant randomised into GROUP A at the beginning of the trial:
Group A QUESTIONS

"All right, thank you! Now, I would like to ask you about your worries."
„Have your concerns been reduced about getting infected with coronavirus?" yes/no
Please rate your concerns now between 1 and 10 (1 being 'it's not typical at all' and 10 being 'it is very much typical')

"Do you fear less for your family because of the coronavirus?" yes/no
Please rate your concerns between 1 and 10 now (1 being 'it's not typical at all' and 10 being 'it is very much typical')

"Do you feel less overwhelmed because of the news about coronavirus? yes/no
Please rate your concerns between 1 and 10 now (1 being 'it's not typical at all' and 10 being 'it is very much typical')

"Finally, I would like to ask you that since our last phone call…
"Do you get more support and help from your family, friends or acquaintances?" yes/no

----

“Thank you, now I would like to ask you about your smoking habits.”
“Since our last call, did you smoke?“

if yes:

“Did you smoke regular cigarettes?”

if yes:

How many cigarettes did you smoke per day?

“Did you smoke an electronic cigarette?”

"In the following, I would like to ask questions about your physical activity:"

"How much time do you spend with physical activity weekly? By this, we mean every gardening, housework, or any physical activity which is longer than 10 minutes.”

(To the operator: this data should be noted in minutes; if the study participant responds in hours, it should be transformed to minutes.)

(To the operator: the definition of physical activity is important; the minimum duration of physical activity should be at least 10 minutes, and includes the following activities: leisure physical activity, physical activity at work, housework, sport, or planned physical activity.)

If the weekly physical activity is less than 150 minutes:

"Do you have any physical disabilities?"

(To the operator: the possible answers are yes or no. Physical disability includes: bedridden, wheelchair bound, amputated limb, stroke.)

“Next I’d like to go through the same line of questions as before about your weight and dietary habits.”
“What is your current weight? (Height is brought on from the form A)
“How much and what kind of fruits do you consume on an average day?”
(To the operator: one portion approximately equals one piece of a bigger fruit (e.g. apples, bananas, oranges) or alternatively one glass of freshly squeezed juice).
“How much and what kind of vegetables do you consume on an average day?”
(To the operator: one portion approximately equals a medium tomato or a bigger pepper. Potato consumption doesn’t count!)
(To the operator: one portion in the case of fruits / vegetables: 150 grams)
“Do you consume meat?

If yes:

“What kind of meats do you consume more frequently: fish – poultry/ beef – pork – lamb-game/ both groups”

“Do you - on an average day - consume any of the following products: soft drinks, pre-packaged snacks (such as frozen pizza and cookies), chocolate or fast food?”

“Thank you. Lastly, I would like to ask about your drinking habits.”
“Have you been drinking alcohol since our last call?”

If yes:

“How often?”

If once a week or more frequently:

"In the last seven days, how much and what kind of alcohol have you consumed?"

If less than once a week:

“How much and what kind of alcohol do you usually consume per occasion?”

If no:

“Since when do you not consume alcohol?”

GROUP A INTERVENTIONS

STRESS GENERAL INTERVENTION:

As far as possible, maintain interpersonal relationships, keep in touch with family friends or acquaintances. Pay attention to your own needs and feelings. Engage yourself in activities that you enjoy and find calming. Try to avoid any information coming from unreliable sources. Mainly rely on information coming from health professionals. Because of the coronavirus related regulations, our social interactions can become limited easily, anybody can feel alone in a situation like this. If you are feeling very alone, please let us know, and we can provide you a phone number that you can call to get professional help. (If the participant asks for the phone number: *Magyar Elsősegély Lelki Szolgálat*: 116-123.)

SMOKING, BMI, PHYSICAL ACTIVITY, ALCOHOL

If the lifestyle is unhealthy in any aspect: Since smoking, physical activity, diet, and alcohol consumption influence the immune functions of your body, we recommend that during the coronavirus pandemic you try to stop smoking / stop drinking alcohol / engage in regular physical activities for at least 150 minutes a week / eat healthy, because these factors may both prevent getting infected with viruses and improve severity of an infection. Alcohol is harmful for the body and doesn’t give protection against coronavirus infection. Upper airway infections are much milder in non-smokers. If you have any questions about these topics, please contact your family doctor for any further information.

Positive feedback: Based on our answers, you are leading a healthy lifestyle. Considering that cessation of smoking and alcohol consumption, regular exercise, and a balanced diet can have a protective effect, we recommend continuing to follow this lifestyle, both because of the likelihood of infection with the virus and the severity of the disease course. In addition, we would like to highlight the importance of drawing attention to these factors during public health and quarantine measures, for example, physical activity can be easily reduced and your weight easily increased. Alcohol is harmful to the body and doesn’t give protection against coronavirus infection. Upper airway infections are much milder in non-smokers. If you have questions about any of these topics, contact your physician.

CLOSING REMARKS:

Thank you for your participation! We hope that we could help you with our advice. For a regular, not just an occasional health improvement, we will call you again in 1 week / 2 weeks / a month. Next time you won’t have to listen to the long information at the beginning of the phone call again. Please don’t put down the phone yet, because we would like to ask you to give us a really short feedback with the help of the buttons. Goodbye!

--

<u>Participant randomised into GROUP B at the beginning of the trial:</u>
<u>Group B QUESTIONS</u>

STRESS QUESTIONS:

"All right, thank you! Now I would like to ask you about your worries."
"Have your concerns decreased about being infected with coronavirus?"
Please rate your concerns now between 1 and 10 (1 being 'it's not typical at all' and 10 being 'very much typical')

"Do you fear for your family less because of the coronavirus?"
Please rate your concerns between 1 and 10 now

"Do you feel less overwhelmed because of the news about coronavirus?"
Please rate your concerns between 1 and 10 now

"Finally, I would like to ask you, since our last phone call…"
"Do you get more support and help from your family, friends or acquaintances?"

STRESS PERSONALIZED INTERVENTION

If any of the questions 1-3 is >4 OR question 4 is NO:
As much as your opportunities allow, keep up your social connections, stay in touch with your family, friends, and acquaintances. If because of coronavirus related regulations you are required to avoid physical contact with others, try maintaining your social connections through other platforms, e.g. telephone, e-mail, social media (Facebook, TikTok, Skype, Twitter, etc.).
Establish a daily rhythm! Pay attention to your own needs and feelings. Engage in activities that you like and that make you feel calm, for example: watching movies or reading.
Share your thoughts with others! At the same time, you also should pay attention to the ones around you, listen to them, encourage them to share their experiences and feelings.
Constantly following news related to the epidemic can make you feel overwhelmed. Do not constantly follow this news, only periodically! Try to avoid all information that is not coming from a reliable source. You should primarily rely on news coming from healthcare professionals, for example the governmental information page: https://koronavirus.gov.hu/. Or dial any of the following informational lines: (06 80 277 455 / 06 80 277 456).
Because of the coronavirus related regulations, our social interactions can become limited easily, anybody can feel alone in a situation like this.
If you are feeling very alone, please let us know, and we can provide you a phone number that you can call to get professional help. (If the participant asks for the phone number: *Magyar Elsősegély Lelki Szolgálat*: 116-123)

If all questions 1-3 are <=4 and question 4 is YES:
Thank you. Maintaining your composure can be very important in situations like this. Having the support of others can help a lot.

--

SMOKING QUESTIONS:

"Thank you, now I would like to ask you about your smoking habits."
"Since our last call, did you smoke?"

if yes:

"Did you smoke regular cigarettes?"

if yes:

How many cigarettes did you smoke per day?

“Did you smoke an electronic cigarette?”

SMOKING PERSONALIZED INTERVENTION:

Stopped smoking completely: “Congratulations, you have taken a very big step for your health since our last call. Keep it up, stay smoke-free. Upper airway infections are milder in non-smokers.”
Cut down smoking: „I see that you smoke less since our last call. You are doing well. Try to stop smoking completely. Upper airway infections are milder in non-smokers.”
Started smoking, smokes the same amount or more: “I see that you started smoking/ you are smoking the same amount/you increased the amount of smoking. Upper airway infections are milder in non-smokers. Accordingly, we can highly recommend that you stop smoking as soon as possible. If you are unable to do this, you might cut down smoking at least. If you need any further support to quit smoking, please let us know now.“
If yes: “To get help with quitting, call 06-80-44-20-44, which is a free service.”
Still doesn’t smoke: „Very good, keep it up and stay smoke-free. Upper airway infections are milder in non-smokers.”

--

PHYSICAL ACTIVITY QUESTIONS:

"In the following I would like to ask questions about your physical activity:"

"How much time do you spend with physical activity weekly? By this, we mean every gardening, housework, or any physical activity which is longer than 10 minutes.”

(To the operator: this data should be noted in minutes; if the study participant responds in hours, it should be transformed to minutes.)

(To the operator: the definition of physical activity is important; the minimum duration of physical activity should be at least 10 minutes, and includes the following activities: leisure physical activity, physical activity at work, housework, sport, or planned physical activity.)

If the weekly physical activity is less than 150 minutes:

"Do you have any physical disabilities?"

(To the operator: the possible answers are yes or no. Physical disability includes: bedridden, wheelchair bound, amputated limb, stroke.)

PHYSICAL ACTIVITY PERSONALIZED INTERVENTION
(In every case, counselling starts with the sentences below, then it continues according to the decision tree.)

Opening sentence: “The most common complications of the coronavirus infection are lung and heart disease, which has a major influence on the need for intensive care. If you perform enough physical activity, it can act as a protective factor against complications. Being home for long periods is a danger in terms of reduced physical activity. We recommend that you try to avoid any close contact with others during exercise because it can increase the risk of infection.”

If the participant exercised more than 300 minutes weekly, and it has been completed again since the last call, POSITIVE FEEDBACK:

“You completed the 300 minutes of physical activity you have mentioned previously, therefore we recommend its continuation.”

If the participant exercised more than 300 minutes weekly, and it has been completed again since the last call, GUIDANCE 7:

“You did not complete the 300 minutes of physical activity you have mentioned previously, so we recommend that you restore it.”

If the participant exercised 150-300 minutes weekly, and it has been increased since the last call, POSITIVE FEEDBACK+GUIDANCE 6:

“You have successfully increased your weekly physical activity, you did great for your health. You can further increase this performance.”

If the participant exercised 150-300 minutes weekly, and it has not been increased since the last call, GUIDANCE 5:

“You did not manage to increase your weekly physical activity. In order to do more for your health, you can further increase your performance."

If the disabled participant has increased weekly physical activity (less than 150 minutes weekly), POSITIVE FEEDBACK + GUIDANCE 4:

"You have done a lot for your health, and we recommend that you continue with this."

If the disabled participant has not increased weekly physical activity (less than 150 minutes weekly), GUIDANCE 3:

“In order to stay healthy, we recommend increasing your physical activity beyond 150 minutes per week. Contact your doctor for more detailed advice."

If the participant has increased weekly pshysical activity (less than 150 minutes weekly), POSITIVE FEEDBACK + GUIDANCE 2:

“You successfully increased your weekly 150 minutes of physical activity that you provided previously, you did a lot for your health. You can further increase this performance."

If the participant (without physical disability) exercised less than 150 minutes weekly, and it has been not increased since the last call, GUIDANCE 1:

“You did not increase your weekly 150 minutes of physical activity that you provided previously. In order to do more for your health, you can further increase this performance."

BMI AND DIETARY QUESTIONS:

“Next I’d like to go through the same line of questions as before about your weight and dietary habits.”
“What is your current weight? (Height is brought on from the form A)
“How many and what kind of fruits do you consume on an average day?”
(To the operator: one portion approximately equals one piece of a bigger fruit (e.g. apples, bananas, oranges) or alternatively one glass of freshly squeezed juice.)
“How many and what kind of vegetables do you consume on an average day?”
(To the operator: one portion approximately equals a medium tomato or a bigger pepper. Potato consumption doesn’t count!)
(To the operator: one portion in the case of fruits / vegetables: 150 grams)
“Do you consume meat?

If yes:

“What kind of meats do you consume more frequently: fish – poultry / beef – pork – lamb-game or both groups?”

“Do you, on an average day consume any of the following products: soft drinks, pre-packaged snacks (such as frozen pizza and cookies), chocolate or fast food?”

<u>BMI AND DIETARY PERSONALIZED INTERVENTION</u>

<u>Beginning sentences:</u> „We’d like to bring to your attention to the fact that an overly low or an overly high body weight poses an increased risk in terms of severity in every disease; thus, it increases the risk of a more severe coronavirus infection. Similarly, unhealthy dietary habits can negatively affect various factors including, for example, the risk of cardiovascular complications. With the current situation - leading to greater time being spent at home - the probability of gaining weight and taking to unhealthy dietary habits is higher.”

<u>Dietary recommendation</u>:
(To the operator: The part where the index questions detected unhealthy consumption should be highlighted for the individuals.)
“We advise that you regularly consume fruits, vegetables, legumes (e.g. beans, peas, lentils), unsalted nuts, and whole grain products (such as millet, oats, wheat and brown rice). You should consume at least 400 grams of vegetables and fruits every day (excluding potatoes!). Free sugars, usually present in soft drinks and sweets are better avoided. Avoid consuming fatty foods, fat intake should amount for less than a third of your daily calorie intake. Products containing unsaturated fats such as fish, nuts, sunflower- and olive oil are preferred, you should limit products containing saturated fats (such as fatty meat, cream, and cheese) and you should avoid products containing trans-fats and additives such any pastries and frozen foods, pre-packed snacks (crisps, cookies), fast-food items, meat of ruminant animals (such as cow).”
“Naturally, if you are receiving dietary advice from a medical professional for health-related reasons, we recommend strict compliance with those recommendations.”

<u>BMI 18.5-29.99</u>: “Compared to your body height, your body weight is ideal with regard to the risk of viral infection.”

<u>BMI <18.5</u>: “Additionally, as your bodyweight is lower than the optimal range for your height, we recommend that you increase your food consumption as much as possible.”

<u>BMI >30</u>: “Additionally, as your body weight exceeds the optimal range for your height, we recommend that you decrease your consumption. It is important that this limitation must not be extreme, which could also pose a threat to your health and safety. We recommend 0.5-1 kg / week weight loss as a target.”

<u>Positive feedback + recommendation</u>: “Based on the data you provided, your weight has improved since the last call, which is great news. As it still hasn’t reached the optimal range, we recommend that you continue losing / gaining weight.” + dietary recommendation (**the same**)!

<u>If formerly unhealthy BMI has reached the target range</u>: “Congratulations you’ve successfully lost / gained weight, you reached the target optimal range. We do not recommend further weight loss / gain.“

<u>Positive feedback:</u> „Your dietary habits, as assessed by our questions, still follow a healthy pattern. Still, we would again like to inform you on the concepts of a healthy diet:
“We advise that you regularly consume fruits, vegetables, legumes (e.g. beans, peas, lentils), unsalted nuts, and whole grain products (such as millet, oats, wheat and brown rice). You

should consume at least 400 grams of vegetables and fruits every day (excluding potatoes!). Free sugars, usually present in soft drinks and sweets are better avoided. Avoid consuming fatty foods, fat intake should amount for less than a third of your daily calorie intake. Products containing unsaturated fats such as fish, nuts, sunflower- and olive oil are preferred, you should limit products containing saturated fats (such as fatty meat, cream, and cheese) and you should avoid products containing trans-fats and additives such any pastries and frozen foods, pre-packed snacks (crisps, cookies), fast-food items, meat of ruminant animals (such as cow)."
"Naturally, if you are receiving dietary advice from a medical professional for health-related reasons, we recommend strict compliance with those recommendations."

ALCOHOL CONSUMPTION QUESTIONS:

"Thank you. Lastly, I would like to ask about your drinking habits."
"Have you been drinking alcohol since our last call?"

If yes:
"How often?"

If once a week or more frequently:
"In the last seven days, how much and what kind of alcohol have you consumed?"

If less than once a week:
"How much and what kind of alcohol do you usually consume per occasion?"

If no:
"Since when do you not consume alcohol?"

ALCOHOL CONSUMPTION PERSONALIZED INTERVENTION:

Continued drinking no alcohol: "I see you still don't drink alcohol. You're doing great. Keep it up! Alcohol is harmful to the body and doesn't give protection against coronavirus infection."
Less alcohol consumption but not abstinent: "We are glad that you drink less alcohol since the last conversation. You are on the right track! Try to stop drinking alcohol completely. Alcohol is harmful to the body and doesn't give protection against coronavirus infection."
Achieved abstinence: "Congratulations, you took a very big step in protecting your health. Maintain your abstinence! Alcohol is harmful to the body and doesn't give protection against coronavirus infection."
Started drinking alcohol or consumes the same amount or more:
(To the operator: highlight what she/he consumes based on self-declaration).
"I see that since our last phone call you started to drink alcohol/ drink more alcohol/ didn't change your alcohol consumption. There are two very important things to know about alcohol consumption: Alcohol consumption is harmful and it does not protect you against the coronavirus.
I advise you to consume as little alcohol as possible and do not exceed the following quantities:
- for men: 3 dL of wine or3 glasses of beer or 6 cL hard drinks
- for women: 2 dL of wine or 2 glasses of beer or 4 cL hard drinks or 2 cocktails
Furthermore, do not consume more than 4 dl of wine or 4 glasses of beer or 8 cL of hard drink per occasion. The best thing you can do for your health is if you do not drink alcohol at all."

CLOSING REMARKS:

“Thank you for your participation! We hope that we could help you with our advice. For a regular, not just an occasional health improvement, we will call you again in 1 week / 2 weeks / a month. Next time you won’t have to listen to the long information at the beginning of the phone call again. Please don’t put down the phone yet, because we would like to ask you to give us a really short feedback with the help of the buttons.
Goodbye!”
